# Extensive Profiling of Polyphenols from Two *Trollius* Species Using a Combination of Untargeted and Targeted Approaches

**DOI:** 10.3390/metabo10030119

**Published:** 2020-03-23

**Authors:** He Tian, Zhiyang Zhou, Guanghou Shui, Sin Man Lam

**Affiliations:** 1 Institute of Genetics and Developmental Biology, Chinese Academy of Sciences, Beijing 100101, China; tianhe@genetics.ac.cn; 2 Lipidall Technologies Company Limited, Changzhou 213022, China; zyzhou@lipidall.com

**Keywords:** polyphenols, globeflowers, flavonoids, mass spectrometry

## Abstract

Various species of globeflowers, belonging to the genus *Trollius*, have been extensively used in traditional Chinese medicine due to their anti-inflammatory, antimicrobial, and antiviral properties, which are mainly attributed to their high polyphenol content. Differences in polyphenol composition, and abundances, will lead to varying treatment efficacies of globeflowers. Herein, we employ a combination of targeted and untargeted mass spectrometry (MS) approaches to characterize and quantify a comprehensive array of polyphenols, mainly including flavonoids and phenolic acids in two globeflower species commonly used in Chinese medicine, *Trollius chinensis* Bunge and *Trollius ledebouri* Reichb. In addition, free radical scavenging activity was investigated to evaluate the association between polyphenol composition and antioxidation capacity. Liquid chromatography (LC)-based separation and multiple-reaction-monitoring (MRM) transitions were optimized using a library of 78 polyphenol reference compounds to achieve absolute quantification on triple quadrupoles MS (QqQ). The analytical method was further expanded via high-resolution MS to provide relative quantitation of an additional 104 endogenous polyphenols in globeflowers not included in our reference library. Our results revealed stark differences in polyphenol content between *T. chinensis* and *T. ledebouri*, emphasizing the need for systematic characterization of polyphenol composition to ensure treatment efficacy and consistency in standardizing the use of globeflowers in Chinese medicine.

## 1. Introduction

The genus *Trollius* comprises 31 species inhabiting the northern hemisphere areas, which have been used in folk medicine in Europe, western Siberia, and China [1]. *Trollius chinensis* Bunge and *Trollius ledebouri* Reichb, mainly produced in northern China [2], are commonly used in Chinese traditional medicine to treat upper respiratory infections, pharyngitis, tonsillitis, esoenteritis, canker, bronchitis, etc. 

Polyphenols, which mainly include flavonoids and phenolic acids [3], are present, in high abundance, amongst the genus *Trollius* (including *T. chinensis* and *T. ledebouri*), and are responsible for the antiviral, antimicrobial, antioxidant activities associated with these plant species [4,5]. Previous research had demonstrated that specific polyphenols impede cancer cell proliferation [6]. A recent study showed that orientin and vitexin exhibited appreciable inhibitory effects on the proliferation of the esophageal cancer (EC)-109 cells [7]. Moreover, orientin displayed higher antitumor effects than vitexin. In another study, the extract from *T. chinensis* displayed a strong inhibitory effect on proliferation of human gastric carcinoma cells, human melanoma cells, and two different cell lines of human breast adenocarcinoma [8,9]. 

Preceding studies on the pharmacological activity globeflowers, however, were mostly confined to only one species of *Trollius*, without comparing the differences among frequently used globeflowers, such as *T. chinensis* and *T. ledebouri* [10,11,12]. In most cases, Chinese medicine practitioners do not differentiate between *T. chinensis* and *T. ledebouri* and consider them as one [2]. As a result, endogenous differences in the compositions of polyphenols between *T. chinensis* and *T. ledebouri* can lead to varying treatment efficacies when they are combined in differing proportions [13]. Thus, a systematic comparison of bioactive components (in particular polyphenols) in *T. chinensis* and *T. ledebouri*, is essential to standardize the use of globeflowers in Chinese medicine to ensure consistency in terms of treatment effects.

Currently, LC-MS is the most widely adopted technique used to characterize and quantify phenolic compounds in various food, plants, and herbs [14,15,16]. With rapid development in tandem mass spectrometry and chromatographic separation techniques, numerous phenolic compounds have been characterized and identified as the primary antioxidants and functional components in various fruits, vegetables, agricultural products, herbs, and plants [14,17,18]. Tandem triple quadrupole (QqQ)-MS is regarded as the gold standard in quantification due to its superior sensitivity and wide dynamic ranges over high-resolution MS (HR-MS) [19]. Nonetheless, QqQ has constraint in terms of compound identification, owing to its limitation in acquiring accurate mass to charge ratio (*m*/*z*) of molecules, making QqQ-MS heavily reliant on the need of reference standards/compounds to achieve unambiguous identification [19]. Considering the plethora of metabolites and bioactive compounds in plants and other biological samples, it is often economically impractical and technically infeasible to obtain all of reference standards to achieve compound identification solely via QqQ-MS. In comparison, HR-MS is powerful in identifying unknown compounds and providing semi-quantitative results. 

Information dependent acquisition (IDA) using HR-MS can provide annotated identification results even in the absence of reference standard compounds [20]. A shortcoming of IDA, however, lies in its limited acquisition of product ion spectra, as the secondary mass spectra of only the top ten to twenty precursor ions can be recorded in a single scan cycle, while parent ions of lower intensities are not triggered for MS/MS. In comparison, data independent acquisition (DIA) simultaneously performs fragmentation of all precursors, albeit resulting in a higher complexity in identification due to composite product ion spectra from co-eluting substances [21]. 

**Figure 1:**
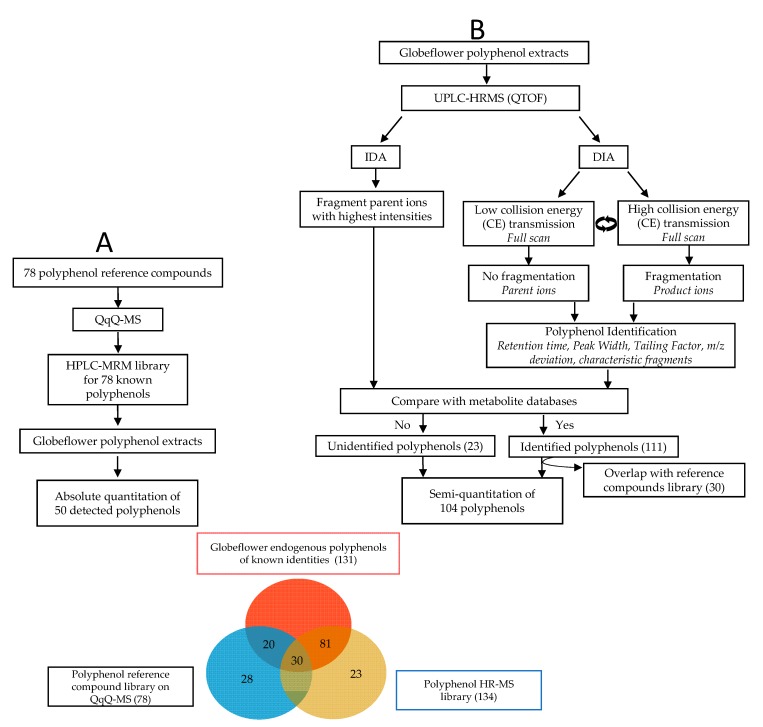
Schematic diagram illustrating combination of (**A**) using HPLC-QqQ (MRM) and (**B**) using UPLC-QTOF (IDA and DIA) approaches for identification and quantification of polyphenols in globeflowers. HPLC, high-performance liquid chromatography; QqQ, tandem triple quadrupole; MS, mass spectrometry; HR, high resolution; UPLC, ultra-performance liquid chromatography; QTOF, tandem quadrupole time of flight.

In this study, we first constructed and optimized a high-performance liquid chromatography (HPLC)-multiple-reaction-monitoring (MRM) method based on QqQ-MS using a library of 78 reference polyphenol standards, which was used to provide absolute quantification of these polyphenols in *T. chinensis* and *T. ledebouri*. Next, we employed HR-MS in both IDA and DIA modes to expand our existing library to include an additional 104 endogenous polyphenols in *T. chinensis* and *T. ledebouri*, of which 81 had product ion spectra that matched with known metabolites in commercial databases (Figure 1). In all, we report the absolute/relative quantitative results of 131 identified and 23 unidentified polyphenols in the two species of globeflowers under investigation. 

## 2. Results

### 2.1. Column Selection for Polyphenol Separation

Waters High Strength Silica (HSS) T_3_ column (2.1 mm × 100 mm, 1.8 µm) and Phenomenex Kinetex C18 (2.1 mm × 100 mm, 1.8 µm) were selected to compare their separation behaviors for polyphenols. Different composition of mobile phases were also investigated, including pure aqueous phase (i.e., water containing 0.1% formic acid; FA) and pure organic phase, i.e., acetonitrile (ACN) or methanol (MeOH), as well as aqueous-organic mixtures including water/ACN or MEOH containing 0.1% FA and 20 mM ammonium acetate, etc. Our results showed that mobile phases comprising water containing 0.1% FA, and pure ACN are the ideal mobile phase combination for both columns, considering the peak shapes of all 78 polyphenol reference compounds (data not shown). The total elution time of HSS T_3_ for the tested reference compounds was shorter and peak shapes were more ideal than that of Kinetex C18 (Figure S1); thus, HSS T_3_ column was chosen due to shorter analytical duration and better chromatographic behaviors.

### 2.2. Identification and Quantification of Polyphenols

An HPLC-MRM method was constructed on QqQ-MS using 78 reference polyphenol standards (Table S1), MRM transitions of individual polyphenols were presented in Table S2. Of these polyphenols, 50 were detected in the extracts of *T. chinensis* and *T. ledebouri*, and standard addition was used to provide absolute quantitation of these 50 polyphenols in globeflowers (Table S3). Furthermore, an additional 104 polyphenols were putatively identified via HR-MS on ultra-performance liquid chromatography coupled to tandem quadrupole time of flight (UPLC-QTOF) and were relatively quantified.

In the IDA method, 20 most intense parent ions in one scan cycle were selected for further fragmentation, and the resultant product spectra were compared with metabolite databases for identification. For the DIA method, parent-product ion pairs, separately generated from experiment 1 (scans for parent ions) and 2 (scans for product ions of all parent ions), were first output into two dimensional matrix, including mass-to-charge ratios (*m/z*), retention time (*rt*), start and end time, and tailing factors of all ion peaks. In the second step, precursor and daughter ions were processed to match with each other according to the following criteria: (i) *rt* (min) should be equal between ion pairs (accurate to two decimal places), (ii) span of start and end time of product ions should be within that of their corresponding parent ions, (iii) tailing factors of product ions should be equal to or less than that of their corresponding parent ions. If an ion pair satisfied all of the above criteria, then evaluation of characteristic fragmentation will be conducted further both for DIA and IDA results to judge whether these ion pairs belong to polyphenols. 

For identification of a parent ion as flavonoid or phenolic acid, it requires matching of three or more product ions that are characteristic fragments of flavonoids and phenolic acids with the parent ion under evaluation (Table 1). For identification of flavonoid glycosides, on top of satisfying the identification criteria for flavonoids or phenolic acids as aforementioned, a parent ion needs to have at least one neutral loss matching with the characteristic neutral loss for flavonoid glycosides (Table 2). Using HR-MS approaches, a total of 134 candidate polyphenols matched the identification criteria mentioned above. Amongst these compounds, 111 polyphenols exhibited product ion spectra that matched with known polyphenols in metabolite database, of which 30 coincided with polyphenols included in our own reference library constructed on QqQ-MS (Figure 1), and 23 polyphenols remained unidentified. Therefore, the current work had (putatively) identified and absolutely/relatively quantified a total of 131 polyphenols endogenous to globeflower extracts.

**Figure 2:**
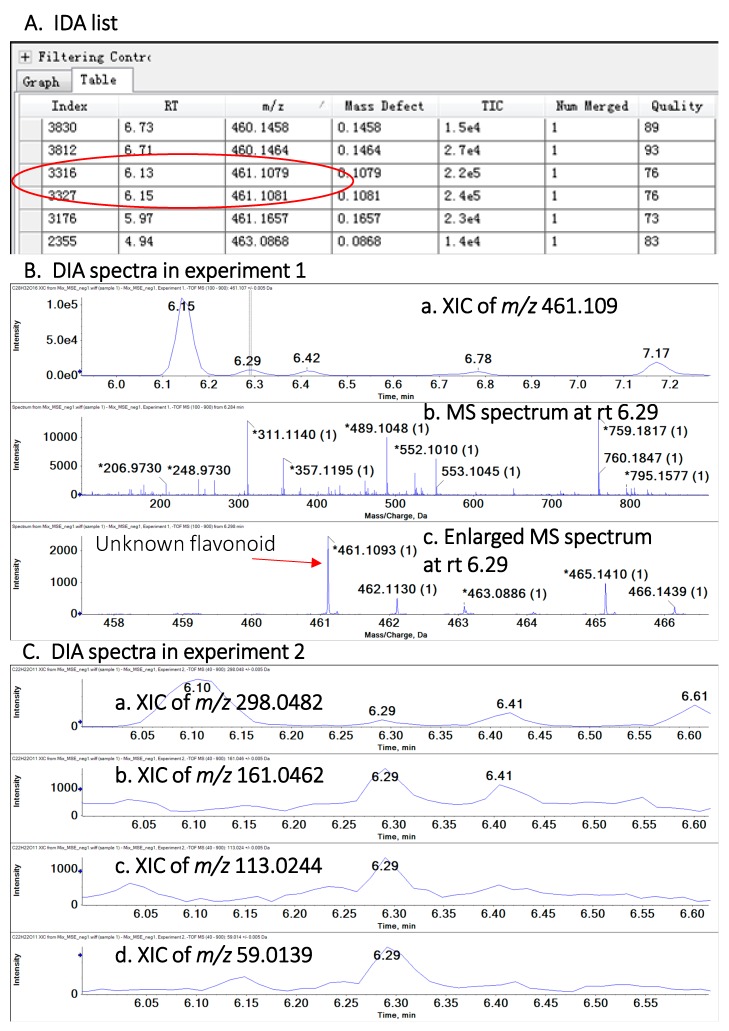
Example illustrating how DIA serves to characterize endogenously low polyphenols missed by IDA scan. (**A**) IDA list showing the acquired results of ions around *m/z* 461.109 and RT 6.29 minutes. (**B**) The XIC of *m/z* 461.109 and its corresponding spectrum an RT 6.29 minutes in DIA experiment 1. (**C**) XIC of product ions (*m/z* 298.0842, *m/z* 161.0462, *m/z* 113.0244, *m/z* 59.0139) of *m/z* 461.109 at RT 6.29 minutes in DIA experiment 2. XIC, extracted ion chromatogram; RT, retention time.

To demonstrate the advantages of combining the use of IDA and DIA in HR-MS, we use the example of putatively identified kaempferide 7-hexoside, an endogenously low polyphenol (*m/z* 461.109) in globeflowers. In the IDA list of this study (Figure 2A), there were two ions at *m/z* 461.1079–461.1081 from index order 3316 to 3327 (circled red), and their corresponding rt were also displayed. Product ion spectra of every parent ions in the IDA list were acquired in the IDA scan. However, DIA results generated an additional ion (*m/z* 461.109, rt 6.29), corresponding to kaempferide 7-glucoside, which was not included in IDA list due to its comparatively low intensity. Figure 2B (panels A, B) illustrate the extracted ion chromatograms (XICs) of ions at rt 6.29 min, of which *m/z* 461.109 was not observed. This ion can be seen when the spectrum was enlarged (Figure 2B, panel C). Since its intensity was lower than other signals eluting within the same retention time window, IDA scan failed fragment this ion, leading to the missing information. By comparison, DIA can fragment all of parent ions, independent of ion intensities. Figure 2C displays the XICs of product ions from the parent ion at *m*/*z* 461.109, rt 6.29 min, which coincided with the characteristic fragmentations of flavonoids (Table 1 and Table 2). In the product ion spectra of *m/z* 461.109, 161.0462 (Table 1, No13), 113.0244 (Table 1, No 24) 59.0139 (Table 1, No 31) corresponded to fragments of flavonoid aglucones, while ion at *m*/*z* 298.0482 represented the fragment after neutral loss of 163.0612 C_6_H_11_O_5_^-^ (Table 2, No 8).

### 2.3. Method Validation

Optimized MRM transitions in QqQ-MS were listed in Table S2. The HPLC-MRM method was validated in terms of dynamic linearity range (DLR), limit of detection (LOD), limit of quantification (LOQ), recovery, as well as relative standard deviation (RSD) across three consecutive days (Table S3). Different concentrations of all 78 standard references solutions were spiked into matrix-matched globeflower extracts for the determination of DLR, LOD, and LOQ of QqQ-MS. Precision is satisfactory, with RSD below 14% across consecutive three days in all three spiked concentrations, except for procyanidin B2 (23.89% at lowest spiked concentration of 625 μg·L^−1^) and resveratrol (16.89% at lowest spiked concentration of 625 μg·L^−1^), LOD and LOQ ranged from 0.12 μg·L^−1^ and %0.40 μg·L^−1^, respectively, for formononetin, to 33.33μg·L^−1^ and 111.11 μg·L^−1^, respectively, for 4-Hydroxy-3,5-dimethoxybenzoic acid). Recovery was within 70–120% for all spiked polyphenol standard references, except for 4-Hydroycoumarin.

### 2.4. Polyphenol Profiles of T. chinensis and T. ledebouri

**Figure 3:**
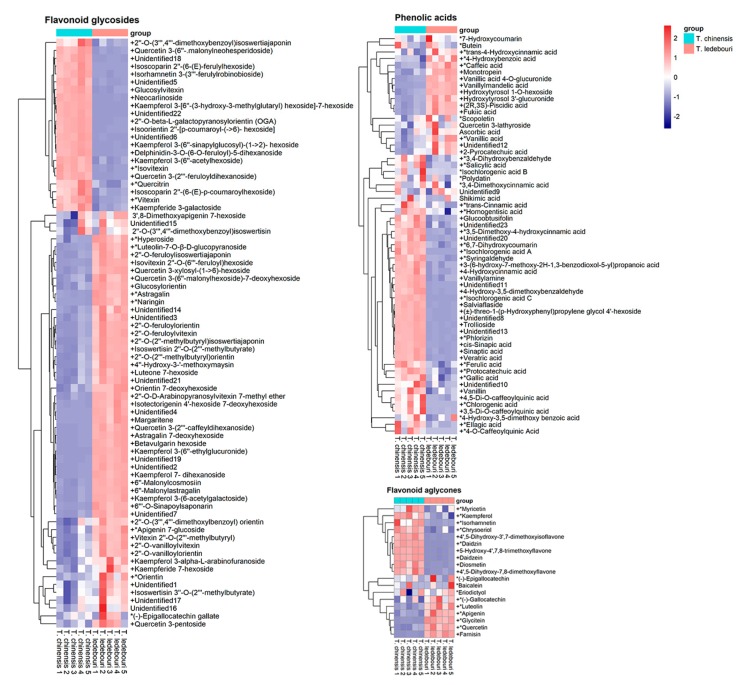
Heatmaps illustrate the differences in levels of flavonoid glycosides, phenolic acids, and flavonoid aglycones between *T. chinensis and T. ledebouri* extracts. The symbol “+” indicates *p* < 0.05, and * for absolutely quantified polyphenols.

**Figure 4:**
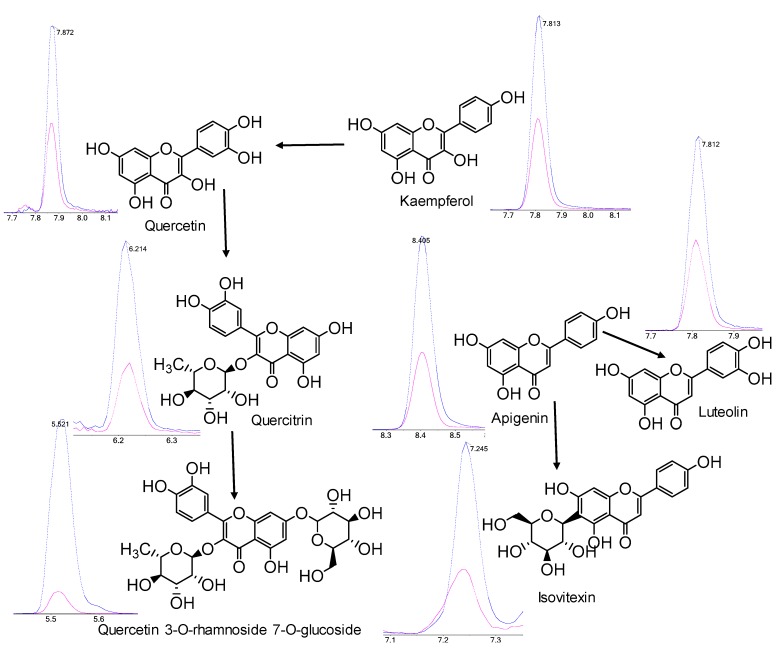
Pathway of flavone and flavonol biosynthesis adapted from the KEGG pathway. Blue line and red line indicate the extracted ion chromatograms for the individual flavonoids in *T. ledebouri* and *T. chinensis*, respectively. KEGG, Kyoto Encyclopedia of Genes and Genomes

In all, 154 polyphenols were quantified (50 absolute quantitation, 104 relative quantitation), including 16 flavonoid aglycones, 80 flavonoid glycosides, 58 phenolic acids (Tables S4 and S5). Among these polyphenols, 135 components were significantly different between *T. chinensis* and *T. ledebouri* (*p* < 0.05), indicating polyphenol biosynthesis are differentially regulated in these two *Trollius* species. The contents of total flavonoid aglycones in *T. chinensis* was 1.63-fold that of *T. ledebouri,* and total phenolic acids in *T. chinensis* was 1.55-fold of *T. ledebouri*. Total flavonoid glycoside in *T. chinensis* was almost equal to those in *T. ledebouri*, but stark differences in flavonoid glycoside composition was observed (Figure 3). Therefore, in general, *T. chinensis* contains significantly higher levels of polyphenols compared to *T. ledebouri*. In particular, *T. ledebouri* exhibited enhanced biosynthesis of flavone and flavonol, with stark elevations in the corresponding pathway bio-constituents compared to *T. chinensis* (Figure 4). In addition, contents of specific phenolic acids, which had been reported to be key bioactive constituents acting against influenza and other viruses [22,23], were found in appreciably higher levels in *T. chinensis* than *T. ledebouri*. For example, isochlorogenic acid C, which exhibits a broad-spectrum antiviral potency against coxsackievirus [24] and human immunodeficiency virus [25], was more than 2000-fold higher in *T. chinensis* than in *T. ledebouri* (Figure 3). These findings aligned with our hypothesis that different *Trollius* species may have different therapeutic potential resulting from their disparate composition of bioactive constituents [2,8,26].

## 3. Discussion

Our present study describes the combined use of QqQ-MS and HR-MS comprising both IDA and DIA methods to achieve high-coverage quantification of polyphenols in two species of globeflowers (*T. chinensis and T. ledebouri*) commonly used in Chinese medicine [1]. With the usage of accurate *m/z* (accurate to four or more decimal places) and relative abundances of parent and product ions, HR-MS is powerful in identifying compounds by matching them with available databases, and further confirmation can be achieved by comparing additional parameters, such as retention time with standard references [19]. Compared with QqQ-MS; however, HR-MS is less suited for quantification due to its limited sensitivity and relatively narrow dynamic ranges, which compromise the repeatability of quantitative data derived from HR-MS [19]. While QqQ-MS operating in the MRM mode is regarded as the gold standard in quantification, it mainly serves to quantify known metabolites, and is reliant on the availability of reference standard compounds to derive optimal transition parameters, including ion source parameters, collision energy, product ions for qualification and quantification, as well as retention time. In most cases, these requirements limit researchers to quantify only known compounds with reference standards in hands.

In biomedical-oriented studies, researchers often hope to obtain quantification results, not only for targeted metabolites, but also for other endogenous metabolites that are structurally similar (i.e., belonging the chemical groups or classes), because these metabolites are expected to elicit similar biological functions or medical properties [27]. Apart from specific metabolite classes, such as lipids, that possess characteristic head groups/fragments that facilitate biochemical classification [28,29,30], such identification and quantification of closely related metabolites without prior known reference standards in hands are extremely challenging for QqQ-MS, which does not come with high mass resolution. Henceforth, we have combined the use of QqQ-MS with HR-MS in the current work to generate more comprehensive polyphenol annotations, and our combined approaches had realized a high-coverage quantification for both identified and unidentified polyphenols in globeflowers. The expansion in metabolite coverage in our work is considerable, even for flavonoid glycosides alone, we have reported a total of 75 species (Table S5) in comparison to a previous work based upon LC/HR-MS on five *Trollius* species that covered only 34 flavonoid glycosides [31]. 

Many of the individual polyphenols, previously reported to be bioactive constituents with notable antiviral potency, are found in significantly different levels between these two species of globeflowers being investigated, suggestive of differing medical efficacy. Further pharmacological experiments are needed to systematically evaluate the bioactivity of antibiosis, antiviral potency, and anti-inflammation capacity among the different *Trollius* species to assure consistency in the medical efficacy of globeflowers. 

## 4. Materials and Methods

### 4.1. Materials

All polyphenol references were obtained from J&K Scientific, Beijing, China. Detailed information of these reference compounds were described in Table S1. Formic acid (FA), was of HPLC grade and purchased from Sigma-Aldrich (Steinheim, Germany). Ultra-pure water (resistivity, 18.2 MΩ) was purified on a Milli-Q Plus apparatus (Millipore, Brussels, Belgium). Acetonitrile (ACN) and methanol (MeOH) of LC-MS grade were purchased from Merck (Darmstadt, Germany). *Trollius chinensis* Bunge and *Trollius ledebouri* Reichb were obtained from Farmer professional cooperative of Corylus heterophylla, JiaGeDaQi district, DaXingAnLing, northwestward of HeiLongJiang Province, China.

### 4.2. Polyphenols Extraction and Preparation

Polyphenols were extracted from Chinese globeflowers, according to previous publications with modifications [14]. Briefly, 20 mg of dried flowers was ground into powder in liquid nitrogen. Next, 0.8 mL of 90% methanol containing 0.5% acetic acid and 0.05% butylated hydroxytoluene was added. Samples were sonicated for 30 min and then centrifuged at 12,000× *g* for 5 min at 4 °C. Clean supernatant was transferred to new tube, and dried using miVac concentrator (Genevac Ltd., Ipswich, UK). Dried extracts were resuspended in 0.5 mL water, containing 0.1% FA, 2% ACN, 0.5 µg of internal standards ((-)-Epigallocatechin, kaempferol-3-O-rutinosid, and resveratrol) for LC-MS analysis.

### 4.3. LC/MSMS

ACQUITY UPLC HSS T_3_ column (2.1 mm × 100 mm × 1.8 µm) and a guard column, both from Waters (Dublin, Ireland), were used. The column oven temperature was maintained at 40 °C, and autosampler was set at 10 °C. The injector volume was 5 µL. Flow rate was 0.40 mL/min. Mobile phase A was water containing 0.1% FA (*v/v*), and mobile phase B was ACN. The following linear gradient was used: 0–1.0 min with 2% B, 1.0–6.0 min with 2%-–42% B, 6.0-8.0 min with 42%–65% B, 8.0–10.0 min with 65%–76% B, 10.0-11.0 min with 76%–100% B, 11.0–14.0 min with 100%–100% B. 

*For the identification of polyphenols*, Agilent 1290 II UPLC coupled to AB Sciex QTOF 5600 Plus was used. The electrospray ionization (ESI) source was set up in positive and negative ion modes, respectively. The MS parameters for detection were: ESI source voltage 5.5 kV or −4.5 kV; vaporizer temperature, 550 °C; drying gas (N_2_) pressure, 60 psi; nebulizer gas (N_2_) pressure, 60 psi; curtain gas (N_2_) pressure, 35 psi; and declustering potential, 80 V. The scan range was *m/z* 100–1000. Data acquisition and processing were performed using Analyst^®^ TF 1.7.1 Software (AB Sciex company, Concord, ON, Canada).

*IDA method*, Information-dependent acquisition mode was used for MS/MS analyses of the polyphenols. The collision energy was set at 35 ± 15 eV. 

*DIA method*, data independent acquisition is composed of two full scan experiment. In first experiment, transmission energy was set at 10 eV or −10 eV to generate precursor ions, and in second experiment, this value was increased to 30 eV or −30 eV to produce product ions. Both experiments ran in full scan modes.

High-resolution MS, isotope abundance ratios, MS/MS, the Human Metabolome database (https://hmdb.ca/), the METLIN database (https://metlin.scripps.edu/), PubChem database (https://pubchem.ncbi.nlm.nih.gov/), a literature search, and standard references were applied to identify ion structures.

*For the quantification of polyphenols*, Japser HPLC system coupled to 4500 MD (AB Sciex company, Concord, ON, Canada) was used. Related MS parameters are listed in Table S2. Due to lack of stable isotope-labeled standards of polyphenols, the method of standard addition was employed to quantify endogenous polyphenols in *Trollius* plants. A series of diluted standard references of polyphenols in different concentrations were added into *Trollius* extract matrix to construct eight-point standard calibration curves. For calibration samples, endogenous polyphenol were deducted from *Trollius* extract matrix without the addition of standard references.

### 4.4. Analysis

Parent and product ions, generated from IDA and DIA method experiments, were extracted using MarkerView 1.3 and MultiQuant 3.0.2 (AB Sciex company, Concord, ON, Canada).

Self-compiled R language program was used to process IDA and DIA data. R 3.6.2, used in this work, was downloaded from open source (https://www.R-project.org/), [32]. For IDA data, the program can determine if the ion pairs belong to polyphenols according to characteristic fragmentations (Table 1 and Table 2). For DIA data, the program first attributes all product ions to their corresponding parent ions with the parameters (rt, peak width, tailing factors), then determines whether they are polyphenols based on characteristic fragmentations.

Standard references, (-)-Epigallocatechin, kaempferol-3-O-rutinosid, and resveratrol, not detected in globeflowers, were used as internal standards to calibrate MS data for quantification. The first standard is aglycones, used for calibration of all flavonoids without glycoside, the second is applied for calibrating all flavonoids with glycoside, and the third is phenolic acid for calibrating all phenolic acid components. In addition, individual contents of those semi-quantified 104 polyphenols were referenced to their corresponding flavonoid aglycones, flavonoid glycosides, or phenolic acids from these three internal standards.

Precision studies were performed according to international guidelines (International Conferenceon Harmonisation, Harmonised Tripartite Guideline, and Validation of Analytical Procedures: Text and Methodology Q2(R1). 1994). Globeflower extracts were spiked with known concentrations (low, middle, and high) of each polyphenol (Table S3). Reproducibility was assessed by inter- and intra-assay coefficient of variation (CV). The inter-assay CV was established by performing 5 assay replicates by three consecutive days. The intra-assay CV was established by 5 replicates. Recovery was calculated on low, medium, and high concentrations of standard references with five parallel replicates. The recovery of the spiked standards was determined by assaying two sets of samples: peak areas of endogenous metabolites were subtracted from those of matrix samples (set 1), and subtracted from those of matrix samples added with standards after preparation (set 2). The recoveries of spiked standards were calculated as the percent ratio of set 1 peak areas to set 2 peak areas.

## 5. Conclusions

Our current study reports the systematic characterization and quantification of polyphenols endogenous to two *Trollius* species, namely *Trollius chinensis* Bunge and *Trollius ledebouri* Reichb. In all, 154 polyphenols (131 identified, 23 unidentified) were profiled and quantified using a combination of HPLC-QqQ-MS and UPLC-HR-MS operating in both IDA and DIA modes. Our findings showed that *T. chinensis and T. ledebouri* are remarkably different in terms of polyphenol content and composition, and contributed new information in terms of standardizing the use of globeflowers in Chinese medicine. Future studies to further evaluate potential differences in other pharmalogical aspects of the *Trollius* species, such as antiviral potency and anti-inflammation capacity, are needed to ensure better consistency in the therapeutic efficacy of globeflowers. Although *Trollius* extracts have been widely and traditionally used in Chinese medicine, large-scale clinical trials to evaluate the therapeutic effects of globeflowers in human cohorts are by far lacking, and formal assessment via randomized, placebo-controlled clinical trials should be conducted in future.

## Figures and Tables

**Table 1:** Characteristic fragments of flavonoids and phenolic acids.

No.	Characteristic Fragments	Theoretical *m/z* [M − H]	No	Characteristic Fragments	Theoretical *m/z* [M − H]
1	C_15_H_10_O_7_	301.0354	18	C_7_H_4_O_3_	135.0088
2	C_15_H_9_O_7_	300.0276	19	C_9_H_10_O	133.0659
3	C_15_H_12_O_6_	287.0561	20	C_5_H_8_O_4_	131.0350
4	C_15_H_10_O_6_	285.0405	21	C_6_H_6_O_3_	125.0244
5	C_14_H_8_O_6_	271.0248	22	C_6_H_4_O_3_	123.0088
6	C_15_H_10_O_5_	269.0455	23	C_7_H_6_O_2_	121.0295
7	C_14_H_8_O_5_	255.0299	24	C_5_H_6_O_3_	113.0244
8	C_13_H_12_O_5_	247.0612	25	C_6_H_4_O_2_	107.0139
9	C_13_H_14_O	185.0972	26	C_5_H_6_O_2_	97.0295
10	C_8_H_6_O_5_	178.9986	27	C_5_H_4_O_2_	95.0139
11	C_12_H_12_O	171.0815	28	C_6_H_4_O	91.0190
12	C_8_H_4_O_4_	163.0037	29	C_7_H_6_	89.0397
13	C_6_H_9_O_10_	161.0456	30	C_4_H_4_O_2_	83.0139
14	C_7_H_4_O_4_	151.0037	31	C_2_H_4_O_2_	59.0139
15	C_8_H_6_O_3_	149.0244	32	C_3_H_4_O	55.0190
16	C_6_H_9_O_4_	144.0428	33	C_3_H_2_O	53.0033
17	C_7_H_6_O_3_	137.0244			

[M − H] refers to deprotonated molecular ion.

**Table 2:** Characteristic neutral loss of flavonoid glycosides.

No.	Formula	Molecular Weight	Molecular Assignment
1	C_7_H_12_O_7_	208.0589	Glucose + CO
2	C_7_H_12_O_6_	192.0640	Glucose + C
3	C_6_H_12_O_6_	180.0628	Glucose
4	C_6_H_10_O_7_	194.0432	Glucuronic acid
5	C_6_H_8_O_6_	176.0326	Glucuronic acid − H_2_O
6	C_4_H_8_O_4_	120.04281	Glucose − C_2_H_4_O_2_
7	C_6_H_10_O_5_	162.0523	Glucose − H_2_O
8	C_6_H_11_O_5_^.^	163.0612	Glucose − HO^.^
9	C_6_H_12_O_5_	164.0679	Rhamnose
10	C_6_H_10_O_4_	146.0574	Rhamnose − H_2_O
11	C_5_H_10_O_5_	150.0523	Arabinose
12	C_5_H_8_O_4_	132.0417	Arabinose − H_2_O or Xylose

## Supplementary Materials

The following are available online at https://www.mdpi.com/2218-1989/10/3/119/s1, Figure S1. XICs of polyphenol reference compounds using different columns, Table S1. Standard references used for quantification, Table S2. MRM parameters used for method validation, Table S3. Summary of the method validation performance characteristics as determined in extracts from Chinese globeflower. Samples (*n* = 5), Table S4. Absolute quantification results of *T. chinensis* and *T. ledebouri* by QqQ-MS. Samples (*n* = 5), Table S5. Quantitative results of 154 polyphenols in *T. chinensis* and *T. ledebouri* extracts.
