# Supplementary material for: Extensive Profiling of Polyphenols from Two Trollius Species Using a Combination of Untargeted and Targeted Approaches
Source: Metabolites. 2020 Mar 23;10(3):119. doi: 10.3390/metabo10030119 (PMC7143900; doi:10.3390/metabo10030119)
Supplement: Supplementary file 1 [file metabolites-10-00119-s001.pdf]

Supplementary Information

Supplementary Figure 1. XICs of polyphenol reference compounds using different columns.

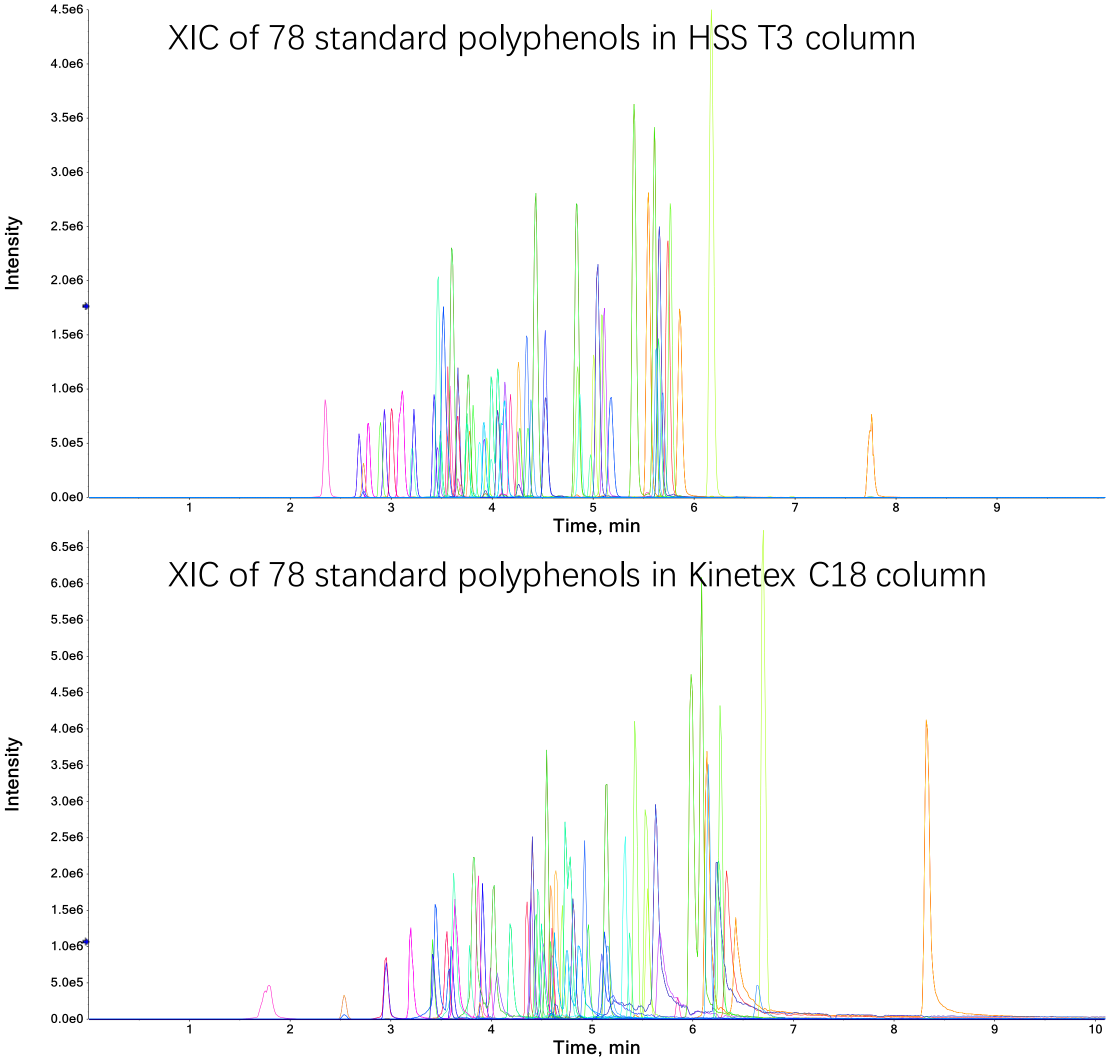

Supplementary Table 1. Standard references used for quantification.

No	References	CAS registry number	Molecular Formula	Purity
1	(+)-Catechin	154-23-4	$C_{15}H_{14}O_6$	99%
2	(-)-Epicatechin	490-46-0	$C_{15}H_{14}O_6$	98%
3	(-)-Gallocatechin	3371-27-5	$C_{15}H_{14}O_7$	98%
4	(-)-Epigallocatechin	970-74-1	$C_{15}H_{14}O_7$	98%
5	(-)-Epicatechin gallate	1257-08-5	$C_{22}H_{18}O_{10}$	99%
6	(-)-Epigallocatechin gallate	989-51-5	$C_{22}H_{18}O_{11}$	98%
7	Kaempferol	520-18-3	$C_{15}H_{10}O_6$	98%
8	trans-4-Hydroxycinnamic acid	501-98-4	$C_9H_8O_3$	98%
9	Vanillic acid	121-34-6	$C_8H_8O_4$	99%
10	Quercetin	117-39-5	$C_{15}H_{10}O_7$	98%
11	4-Hydroxybenzoic acid	99-96-7	$C_7H_6O_3$	99%
12	Ferulic acid	1135-24-6	$C_{10}H_{10}O_4$	99%
13	Chlorogenic acid	327-97-9	$C_{16}H_{18}O_9$	98%
14	4-O-Caffeoylquinic Acid	905-99-7	$C_{16}H_{18}O_9$	98%
15	Neochlorogenic acid	906-33-2	$C_{16}H_{18}O_9$	98%
16	4-Hydroxy-3,5-dimethoxybenzoic acid	530-57-4	$C_9H_{10}O_5$	98%
17	Gallic acid	149-91-7	$C_7H_6O_5$	99%
18	Caffeic acid	331-39-5	$C_9H_8O_4$	99%
19	trans-Cinnamic acid	140-10-3	$C_9H_8O_2$	99%
20	3,5-Dimethoxy-4-hydroxycinnamic acid	530-59-6	$C_{11}H_{12}O_5$	98%
21	2,5-Dihydroxybenzoic acid	490-79-9	$C_7H_6O_4$	99%
22	Apigenin	520-36-5	$C_{15}H_{10}O_5$	98%
23	Protocatechuic acid	99-50-3	$C_7H_6O_4$	99%
24	Rutin	153-18-4	$C_{27}H_{30}O_{16}$	98%
25	7-Hydroxycoumarin	93-35-6	$C_9H_6O_3$	98%
26	Luteolin	491-70-3	$C_{15}H_{10}O_6$	96%
27	Isorhamnetin	480-19-3	$C_{16}H_{12}O_7$	98%
28	6,7-Dihydroxycoumarin	305-01-1	$C_9H_6O_4$	98%
29	Baicalein	491-67-8	$C_{15}H_{10}O_5$	98%
30	(±)-Naringenin	67604-48-2	$C_{15}H_{12}O_5$	98%
31	Apigenin 7-glucoside	578-74-5	$C_{21}H_{20}O_{10}$	98%

32	Isovitexin	38953-85-4	$C_{21}H_{20}O_{10}$	98%
33	Kaempferol-3-O-rutinosid	17650-84-9	$C_{27}H_{30}O_{15}$	98%
34	Astragalin	480-10-4	$C_{21}H_{20}O_{11}$	98%
35	Syringaldehyde	134-96-3	$C_9H_{10}O_4$	98%
36	(+)-Taxifolin	480-18-2	$C_{15}H_{12}O_7$	98%
37	Homogentisic Acid	451-13-8	$C_8H_8O_4$	97%
38	Emodin	518-82-1	$C_{15}H_{10}O_5$	95%
39	Orientin	28608-75-5	$C_{21}H_{20}O_{11}$	98%
40	Glycitein	40957-83-3	$C_{16}H_{12}O_5$	98%
41	Daidzin	552-66-9	$C_{21}H_{20}O_9$	98%
42	Genistin	529-59-9	$C_{21}H_{20}O_{10}$	99%
43	Formononetin	485-72-3	$C_{16}H_{12}O_4$	98%
44	Naringin	10236-47-2	$C_{27}H_{32}O_{14}$	98%
45	Eriodictyol	552-58-9	$C_{15}H_{12}O_6$	98%
46	Enterodiol(P)	80226-00-2	$C_{18}H_{22}O_4$	95%
47	Hesperetin	520-33-2	$C_{16}H_{14}O_6$	98%
48	Nobiletin	478-01-3	$C_{21}H_{22}O_8$	98%
49	Tangeretin	481-53-8	$C_{20}H_{20}O_7$	98%
50	Bergenin	477-90-7	$C_{14}H_{16}O_9$	99%
51	Butein	487-52-5	$C_{15}H_{12}O_5$	98%
52	Phlorizin	60-81-1	$C_{21}H_{24}O_{10}$	95%
53	4-Hydroxycoumarin	1076-38-6	$C_9H_6O_3$	98%
54	Scopoletin	92-61-5	$C_{10}H_8O_4$	98%
55	Daphnetin	486-35-1	$C_9H_6O_4$	98%
56	Chrysoeriol	491-71-4	$C_{16}H_{12}O_6$	95%
57	Luteolin-7-O-β-D-glucopyranoside	5373-11-5	$C_{21}H_{20}O_{11}$	98%
58	Hyperoside	482-36-0	$C_{21}H_{20}O_{12}$	98%
59	3,4-Dimethoxycinnamic acid	2316-26-9	$C_{11}H_{12}O_4$	99%
60	Methyl gallate	99-24-1	$C_8H_8O_5$	99%
61	Procyanidin B2	29106-49-8	$C_{30}H_{26}O_{12}$	98%
62	Hesperidin	520-26-3	$C_{28}H_{34}O_{15}$	98%
63	Quercitrin	522-12-3	$C_{21}H_{20}O_{11}$	98%
64	Resveratrol	501-36-0	$C_{14}H_{12}O_3$	98%
65	Ellagic acid	476-66-4	$C_{14}H_6O_8$	98%
66	Salicylic acid	69-72-7	$C_7H_6O_3$	99%

67	Isochlorogenic acid A	2450-53-5	$C_{25}H_{24}O_{12}$	98%
68	Isochlorogenic acid B	14534-61-3	$C_{25}H_{24}O_{12}$	98%
69	Isochlorogenic acid C	32451-88-0	$C_{25}H_{24}O_{12}$	98%
70	Engenol	97-53-0	$C_{10}H_{12}O_2$	98%
71	Matairesinol	580-72-3	$C_{20}H_{22}O_6$	95%
72	Baicalin	21967-41-9	$C_{21}H_{18}O_{11}$	98%
73	Amentoflavone	1617-53-4	$C_{30}H_{18}O_{10}$	98%
74	Polydatin	65914-17-2	$C_{20}H_{22}O_8$	98%
75	Fisetin	528-48-3	$C_{15}H_{10}O_6$	98%
76	Rosmarinic acid	20283-92-5	$C_{18}H_{16}O_8$	99%
77	3,4-Dihydroxybenzaldehyde	139-85-5	C7H6O3	98%
78	Myricetin	529-44-2	$C_{15}H_{14}O_9$	98%

Supplementary Table 2. MRM parameters used for method validation.

Compound	RT (min)	Ion mode	Parent ion(m/z)	Product ion 1 (m/z)	Product ion 2 (m/z)
flavonoid	2.26	ESI-	366	114	208
(+)-Catechin	5.14	ESI-	289	203	245
(-)-Epicatechin	5.46	ESI-	289	245	203
(-)-Gallocatechin	4.4	ESI-	305	125	179
(-)-Epigallocatechin	4.82	ESI-	305	125	219
(-)-Epicatechin gallate	6.12	ESI-	441	169	289
(-)-Epigallocatechin gallate	5.47	ESI-	457	125	169
Kaempferol	8.3	ESI-	285	117	93
trans-4-Hydroxycinnamic acid	6.21	ESI-	163	119	93
Vanillic acid	5.61	ESI-	167	108	123
Quercetin	7.65	ESI-	301	151	179
4-Hydroxybenzoic acid	5.33	ESI-	137	93	108
Ferulic acid	6.43	ESI-	193	134	117
Chlorogenic acid	5.06	ESI-	353	191	179
4-O-Caffeoylquinic Acid	5.1	ESI-	353	173	179
Neochlorogenic acid	4.6	ESI-	353	191	179
4-Hydroxy-3,5-dimethoxy benzoic acid	5.67	ESI-	197	121	153
Gallic acid	3.74	ESI-	169	125	79
Caffeic acid	5.52	ESI-	179	135	107

**trans-Cinnamic acid**	8.08	ESI-	147	103	119
**3,5-Dimethoxy-4-hydroxycinnamic acid**	6.39	ESI-	223	149	164
**2,5-Dihydroxybenzoic acid**	5.4	ESI-	153	108	109
**Apigenin**	8.18	ESI-	269	117	151
**Protocatechuic acid**	4.6	ESI-	153	109	108
**Rutin**	5.82	ESI-	609	300	255
**7-Hydroxycoumarin**	6.53	ESI-	161	133	105
**Luteolin**	7.58	ESI-	285	133	151
**Isorhamnetin**	8.4	ESI-	315	300	151
**6,7-Dihydroxycoumarin**	5.52	ESI-	177	133	105
**Baicalein**	8.53	ESI-	269	139	167
**(±)-Naringenin**	8.21	ESI-	271	151	119
**Apigenin 7-glucoside**	6.45	ESI-	431	268	240
**Isovitexin**	5.92	ESI-	431	311	341
**Kaempferol-3-O-rutinosid**	6.11	ESI-	593	285	255
**Astragalin**	6.35	ESI-	447	255	284
**Syringaldehyde**	6.46	ESI-	181	151	166
**(+)-Taxifolin**	6.46	ESI-	303	285	125
**Homogentisic Acid**	4.24	ESI-	167	123	122
**Emodin**	10.5	ESI-	269	245	241
**Orientin**	5.66	ESI-	447	327	357
**Glycitein**	7.55	ESI-	283	268	240
**Daidzin**	5.66	ESI+	417	255	
**Genistin**	6.24	ESI+	433	271	
**Formononetin**	8.84	ESI-	267	252	223
**Naringin**	6.33	ESI-	579	271	151
**Eriodictyol**	7.58	ESI-	287	135	151
**Enterodiol(P)**	7.37	ESI-	301	253	271

**Hesperetin**	8.39	ESI-	301	164	151
**Sinensetin**	9.07	ESI+	373	343	321
**Nobiletin**	9.5	ESI+	403	373	388
**Tangeretin**	9.99	ESI+	373	343	358
**Bergenin**	4.8	ESI-	327	192	207
**Butein**	8.02	ESI-	271	135	253
**Phlorizin**	6.7	ESI-	435	273	125
**4-Hydroxycoumarin**	7.18	ESI-	161	117	
**Scopoletin**	6.51	ESI-	191	176	148
**Daphnetin**	5.75	ESI-	177	121	93
**Chrysoeriol**	8.31	ESI-	299	284	256
**Luteolin-7-O-β-D-glucopyranoside**	6.03	ESI-	447	285	327
**Hyperoside**	5.99	ESI-	463	300	271
**3,4-Dimethoxycinnamic acid**	7.41	ESI-	207	103	163
**Methyl gallate**	5.31	ESI-	183	124	140
**Procyanidin B2**	5.18	ESI-	577	289	407
**Hesperidin**	6.42	ESI-	609	301	
**Quercitrin**	6.4	ESI-	447	300	151
**Resveratrol**	7.25	ESI-	227	143	185
**Ellagic acid**	6.04	ESI-	301	145	200
**Salicylic acid**	7.42	ESI-	137	93	65
**Isochlorogenic acid A**	6.39	ESI-	515	353	191
**Isochlorogenic acid B**	6.2	ESI-	515	353	173
**Isochlorogenic acid C**	6.47	ESI-	515	353	173
**Matairesinol**	8.28	ESI-	357	83	122
**Baicalin**	6.91	ESI-	445	269	113
**Amentoflavone**	8.44	ESI-	537	375	417
**Polydatin**	6.02	ESI-	389	227	161
**Fisetin**	6.89	ESI-	285	135	121
**Rosmarinic acid**	6.68	ESI-	359	161	197

**3,4-Dihydroxybenzaldehyde**	5.23	ESI-	137	108	81
**Myricetin**	6.85	ESI-	317	137	179

Supplementary Table 3. Summary of the method validation performance characteristics as determined in extracts from Chinese globeflower. Samples (n=5)

Compound	Calibration equation (y=)	Coefficient of determination	Dynamic range (μg L⁻¹)	LOD (μg L⁻¹)	LOQ (μg L⁻¹)	Spiked (μg kg⁻¹)	Recovery (%)	consecutive 3 days (%RSD)
(+)-Catechin	y = 1.18980e6 x + 15100.40088	0.99629	5-2500	8.00	26.67	625	89.6%	8.1%
						2500	94.7%	5.3%
						6250	93.9%	9.4%
(-)-Epicatechin	y = 4.60455e6 x + 1.01712e5	0.99494	0.25-1000	6.04	20.12	625	83.5%	8.6%
						2500	97.9%	5.8%
						6250	85.8%	4.9%
(-)-Gallocatechin	y = 3.23696e6 x + -1.68322e5	0.99727	10-2500	15.87	52.91	625	96.6%	7.0%
						2500	100.0%	9.2%
						6250	85.3%	6.4%
(-)-Epigallocatechin	y = 3.31448e6 x + -5.31080e5	0.99276	100-2500	6.59	21.98	625	75.1%	8.3%
						2500	84.0%	4.1%
						6250	89.8%	6.7%
(-)-Epicatechin gallate	y = 5.81385e6 x + 2026.20720	0.99864	1-1000	2.99	9.95	625	92.0%	7.8%
						2500	83.0%	7.0%
						6250	89.5%	5.5%
(-)-Epigallocatechin gallate	y = 2.01143e6 x + -2.31861e5	0.99257	5-2500	6.64	22.12	625	92.1%	8.7%
						2500	93.2%	9.7%
						6250	89.4%	6.0%

Kaempferol	y = 8.42601e5 x + -14668.82708	0.99941	1-2500	5.88	19.61	625	91.0%	11.7%
						2500	89.0%	3.1%
						6250	99.0%	5.3%
trans-4-Hydroxycinnamic acid	y = 1.48341e7 x + 1.91271e5	0.9971	10-250	7.26	24.21	625	103.1%	6.0%
						2500	90.7%	3.8%
						6250	94.3%	6.3%
Vanillic acid	y = 3.12050e6 x + 6743.12295	0.99966	0.5-500	6.90	22.99	625	89.6%	7.6%
						2500	90.9%	6.3%
						6250	89.8%	4.4%
Quercetin	y = 8.28497e6 x + -4.57391e5	0.99036	25-2500	1.93	6.42	625	97.8%	8.2%
						2500	90.8%	7.1%
						6250	97.6%	4.5%
4-Hydroxybenzoic acid	y = 3.33790e7 x + 3.57807e5	0.99645	0.25-250	4.48	14.95	625	97.8%	8.0%
						2500	94.5%	6.5%
						6250	86.1%	5.2%
Ferulic acid	y = 1.32026e7 x + 9.76408e4	0.99582	0.1-500	3.44	11.48	625	104.8%	5.5%
						2500	99.9%	5.6%
						6250	99.5%	5.2%
Chlorogenic acid	y = 1.56478e7 x + 4.10184e5	0.99934	1-2500	1.79	5.98	625	94.1%	5.8%
						2500	93.4%	5.3%
						6250	91.9%	5.3%
4-O-Caffeoylquinic Acid	y = 8.48070e6 x + 1.29841e5	0.99943	0.25-2500	2.32	7.73	625	96.6%	5.9%
						2500	99.6%	4.7%
						6250	91.4%	3.6%
Neochlorogenic acid		0.99814	0.5-500	6.32	21.05	625	118.6%	6.1%

	y = 1.07471e7 x + 8.06889e4					2500	102.8%	5.4%
						6250	92.2%	5.1%
4-Hydroxy-3,5-dimethoxybenzoic acid	y = 6.16434e5 x + 2182.63860	0.99848	5-2500	33.33	111.11	625	86.2%	6.4%
						2500	104.3%	7.3%
						6250	93.2%	6.3%
Gallic acid	y = 2.09978e7 x + 25636.96090	0.99328	2.5-1000	1.24	4.12	625	104.7%	6.2%
						2500	96.3%	6.2%
						6250	98.6%	3.2%
Caffeic acid	y = 3.17533e7 x + 4.91710e5	0.99793	2.5-500	4.34	14.47	625	99.0%	4.3%
						2500	93.5%	4.0%
						6250	102.1%	3.3%
trans-Cinnamic acid	y = 1.67166e6 x + 2.56305e5	0.99632	2.5-2500	8.13	27.10	625	82.3%	9.2%
						2500	98.6%	5.4%
						6250	106.9%	6.2%
3,5-Dimethoxy-4-hydroxycinnamic acid	y = 3.34826e6 x + 3.71588e4	0.99788	2.5-1000	5.61	18.69	625	106.2%	2.1%
						2500	102.7%	3.7%
						6250	102.8%	3.0%
2,5-Dihydroxybenzoic acid	y = 2.05251e7 x + 3.03349e5	0.99702	0.25-1000	5.22	17.39	625	87.2%	5.7%
						2500	95.6%	7.5%
						6250	94.0%	6.0%
Apigenin	y = 1.52282e7 x + 2.26293e5	0.99718	0.1-1000	2.33	7.78	625	102.5%	8.4%
						2500	103.4%	6.4%
						6250	103.4%	6.7%
Protocatechuic acid	y = 4.81725e7 x + 8.56361e4	0.99897	1-100	3.81	12.69	625	100.4%	5.3%
						2500	95.5%	6.8%

						6250	97.2%	6.1%
Rutin	y = 2.14215e7 x + 1.00955e5	0.99886	0.1-500	5.08	16.95	625	96.5%	6.5%
						2500	94.3%	6.3%
						6250	93.1%	5.6%
7-Hydroxycoumarin	y = 3.44335e7 x + 2.02994e5	0.9942	0.25-250	7.32	24.39	625	99.8%	6.3%
						2500	92.1%	4.5%
						6250	102.1%	5.2%
Luteolin	y = 1.08559e7 x + 2.45213e5	0.99892	0.25-2500	2.02	6.73	625	95.4%	6.1%
						2500	95.0%	6.4%
						6250	97.5%	3.5%
Isorhamnetin	y = 1.80843e7 x + - 1.25728e6	0.99734	10-2500	6.06	20.20	625	102.7%	3.6%
						2500	99.4%	5.1%
						6250	105.3%	5.3%
6,7-Dihydroxycoumarin	y = 2.70960e7 x + 5.88233e4	0.99776	1-100	4.27	14.22	625	98.0%	4.9%
						2500	101.8%	2.7%
						6250	100.8%	2.5%
Baicalein	y = 1.87874e6 x + - 7.85865e4	0.99087	0.5-2500	5.41	18.02	625	101.9%	3.1%
						2500	96.3%	8.0%
						6250	98.3%	2.8%
(±)-Naringenin	y = 2.53540e7 x + 5.40825e4	0.99956	0.5-250	1.43	4.77	625	95.6%	4.7%
						2500	79.4%	11.2%
						6250	90.2%	3.2%
Apigenin 7-glucoside	y = 1.77909e7 x + 1.62807e5	0.99892	0.1-1000	2.36	7.87	625	92.4%	3.3%
						2500	95.8%	2.1%
						6250	98.9%	4.6%

Isovitexin	y = 1.31684e7 x + 1.73428e5	0.99775	0.5-1000	1.85	6.17	625	93.7%	3.0%
						2500	101.4%	2.8%
						6250	101.0%	2.2%
Kaempferol-3-O-rutinosid	y = 1.48482e7 x + 10799.21249	0.99982	0.25-500	3.42	11.39	625	94.0%	8.6%
						2500	99.3%	8.5%
						6250	103.8%	4.7%
Astragalin	y = 2.50862e7 x + 6.04243e4	0.99904	0.1-500	2.61	8.70	625	97.2%	4.8%
						2500	97.4%	3.8%
						6250	106.7%	3.7%
Syringaldehyde	y = 2.39685e6 x + 22827.46439	0.99876	2.5-1000	2.05	6.85	625	94.1%	9.5%
						2500	109.5%	3.8%
						6250	94.3%	7.2%
(+)-Taxifolin	y = 1.76750e7 x + 1.24435e5	0.99971	2.5-500	2.08	6.93	625	95.1%	9.7%
						2500	92.4%	6.3%
						6250	95.6%	5.3%
Homogentisic Acid	y = 1.12228e7 x + 1.99863e5	0.998	1-1000	6.04	20.12	625	105.2%	4.7%
						2500	101.6%	4.5%
						6250	102.2%	6.5%
Orientin	y = 2.25705e7 x + 2308.97107	0.99967	0.1-250	3.24	10.80	625	101.9%	2.0%
						2500	100.4%	2.4%
						6250	101.3%	2.6%
Glycitein	y = 2.16660e7 x + 4.31584e4	0.99863	0.1-250	3.65	12.18	625	87.1%	5.7%
						2500	100.2%	7.1%
						6250	93.8%	6.0%
Daidzin		0.99953	0.1-1000	4.64	15.46	625	100.6%	2.8%

	y = 2.72009e6 x + 19544.37945					2500	101.0%	2.6%
						6250	100.6%	1.4%
Genistin	y = 9.15247e6 x + 22598.15980	0.9998	0.1-1000	26.55	88.50	625	87.2%	4.5%
						2500	98.7%	4.2%
						6250	86.8%	3.5%
Formononetin	1.42034e8 x + 3.10931e5	0.9965	0.1-100	0.12	0.40	625	117.6%	7.7%
						2500	91.4%	5.1%
						6250	101.0%	5.6%
Naringin	y = 7.82169e6 x + 3.65724e4	0.99878	0.5-500	4.67	15.55	625	81.2%	7.1%
						2500	93.7%	6.7%
						6250	102.8%	6.9%
Eriodictyol	y = 3.10832e7 x + 1.03703e5	0.99884	0.5-250	1.86	6.19	625	94.6%	4.1%
						2500	92.5%	6.4%
						6250	98.1%	6.6%
Enterodiol(P)	y = 2.62915e7 x + - 611.72541	0.99913	0.25-100	3.32	11.07	625	91.4%	8.5%
						2500	95.9%	6.5%
						6250	96.1%	3.8%
Hesperetin	1.81795e7 x + 3.23384e4	0.99979	0.5-250	1.20	4.00	625	95.3%	9.5%
						2500	95.9%	7.1%
						6250	105.3%	4.0%
Bergenin	y = 5.68259e6 x + 11045.65442	0.99931	0.1-500	8.29	27.62	625	97.9%	8.6%
						2500	95.5%	5.0%
						6250	113.1%	4.4%
Butein	y = 2.17637e7 x + - 1.57406e6	0.99582	5-2500	0.92	3.06	625	112.4%	5.5%
						2500	100.8%	4.8%

						6250	106.3%	2.6%
Phlorizin	y = 2.13330e7 x + 7.42339e4	0.99916	0.1-500	3.31	11.03	625	95.8%	7.6%
						2500	97.9%	6.6%
						6250	99.5%	3.2%
4-Hydroxycoumarin	y = 1.12737e8 x + 8.96716e4	0.99682	0.1-50	4.86	16.21	625	43.8%	8.8%
						2500	42.9%	11.3%
						6250	46.4%	6.7%
Scopoletin	y = 3.82756e7 x + 4.67278e4	0.99817	0.1-100	12.05	40.16	625	84.5%	7.8%
						2500	99.1%	8.0%
						6250	94.1%	7.7%
Daphnetin	y = 7.58185e6 x + 1.14556e5	0.99744	2.5-1000	4.43	14.77	625	89.0%	9.0%
						2500	98.8%	4.5%
						6250	95.7%	5.3%
Chrysoeriol	y = 6.04170e7 x + 6.70617e4	0.99885	0.1-100	8.24	27.47	625	104.9%	4.8%
						2500	98.6%	4.5%
						6250	93.8%	4.6%
Luteolin-7-O-β-D-glucopyranoside	y = 1.88754e7 x + 1.29246e5	0.99938	0.1-1000	26.55	88.50	625	98.5%	3.4%
						2500	103.2%	4.3%
						6250	101.6%	2.6%
Hyperoside	y = 1.37714e7 x + 9.61943e4	0.99908	0.25-1000	3.17	10.56	625	100.2%	4.0%
						2500	109.0%	3.5%
						6250	109.1%	2.5%
3,4-Dimethoxycinnamic acid	y = 2.04161e6 x + - 13303.63319	0.99831	1-500	22.39	74.63	625	90.4%	4.9%
						2500	95.0%	8.8%
						6250	102.4%	8.6%

Methyl gallate	y = 3.67764e7 x + 29514.26391	0.99913	1-100	3.95	13.18	625	107.0%	7.6%
						2500	94.3%	9.1%
						6250	94.4%	6.4%
Procyanidin B2	y = 3.42078e6 x + - 8.03870e4	0.99614	1-1000	12.77	42.55	625	72.4%	23.9%
						2500	86.7%	7.6%
						6250	79.9%	7.1%
Hesperidin	y = 1.51841e7 x + 4913.09960	0.99972	0.1-250	6.40	21.32	625	94.5%	4.8%
						2500	90.9%	6.0%
						6250	92.6%	4.9%
Quercitrin	y = 1.98496e7 x + 8.57720e4	0.99885	0.25-500	2.35	7.84	625	103.8%	5.2%
						2500	99.4%	4.3%
						6250	98.1%	4.5%
Resveratrol	y = 5.69155e6 x + 27911.01407	0.9994	0.5-1000	6.70	22.32	625	78.5%	16.9%
						2500	87.8%	13.8%
						6250	87.8%	13.8%
Ellagic acid	y = 1.67751e6 x + - 4.18737e5	0.9638	50-2500	1.72	5.75	625	83.0%	6.4%
						2500	79.8%	4.1%
						6250	101.7%	7.4%
Salicylic acid	y = 6.04396e7 x + 4.42332e5	0.99657	0.5-250	1.78	5.92	625	79.7%	4.8%
						2500	75.9%	5.7%
						6250	92.2%	4.6%
Isochlorogenic acid A	y = 8.85809e6 x + - 14750.74487	0.99909	0.1-1000	0.53	1.77	625	77.8%	5.1%
						2500	97.3%	2.1%
						6250	83.5%	5.8%
Isochlorogenic acid B		0.9992	0.1-2500	1.71	5.70	625	71.2%	4.9%

	y = 5.89359e6 x + 1.05944e5					2500	104.4%	8.1%
						6250	93.1%	4.4%
Isochlorogenic acid C	y = 9.89023e6 x + 1.14082e5	0.99843	0.1-2500	3.72	12.39	625	101.0%	6.5%
						2500	104.5%	5.5%
						6250	108.1%	9.5%
Matairesinol	y = 5.54232e6 x + 2.60055e5	0.99344	10-1000	3.57	11.89	625	107.0%	10.3%
						2500	94.2%	5.3%
						6250	101.5%	6.9%
Baicalin	y = 1.67040e7 x + 7.22858e4	0.99924	1-500	5.41	18.02	625	88.6%	4.1%
						2500	84.2%	5.2%
						6250	89.0%	4.7%
Polydatin	y = 1.13366e7 x + 27670.68243	0.99929	0.25-500	4.59	15.29	625	92.0%	5.9%
						2500	99.2%	4.8%
						6250	99.1%	6.8%
Fisetin	y = 5.50156e6 x + - 1.56017e5	0.99449	10-2500	4.17	13.91	625	102.6%	7.8%
						2500	80.1%	4.3%
						6250	92.8%	7.8%
Rosmarinic acid	y = 1.20570e7 x + - 3.66961e4	0.99972	0.5-1000	2.07	6.89	625	97.8%	8.7%
						2500	84.2%	5.0%
						6250	94.7%	5.5%
3,4-Dihydroxybenzaldehyde	y = 1.78216e7 x + 25896.86562	0.99843	0.25-100	2.35	7.83	625	85.2%	7.6%
						2500	93.7%	11.9%
						6250	94.6%	8.7%
Myricetin	y = 2.66264e6 x + - 2.60900e5	0.98196	0.1-2500	5.29	17.64	625	113.3%	6.9%
						2500	102.0%	7.1%

	6250	102.0%	4.5%

Supplementary Table 4. Absolute quantification results of *T. chinensis* and *T. ledebouri* by QqQ-MS. Samples (n=5),

SampleID	*T. chinensis* (μg/g)					*T. ledebouri* (μg/g)					p value	Fold changes(*T. chinensis* / *T. ledebouri*)
	1	2	3	4	5	1	2	3	4	5		
(-)-Gallocatechin	1.51	1.71	1.53	1.78	1.48	1.73	1.81	1.96	1.65	1.80	4.62E-02	0.90
(-)-Epigallocatechin	4.20	4.23	4.18	4.27	4.17	4.19	4.42	4.18	4.15	4.34	4.32E-01	0.99
(-)-Epigallocatechin gallate	3.12	3.16	3.21	3.16	3.21	3.13	3.35	3.25	3.24	3.13	3.17E-01	0.98
Kaempferol	1.55	1.54	1.68	1.31	1.50	1.11	0.98	0.93	1.12	0.75	3.34E-04	1.55
trans-4-Hydroxycinnamic acid	3.53	2.48	3.97	2.30	2.55	3.72	5.27	3.65	3.90	4.15	2.95E-02	0.72
Vanillic acid	13.53	11.71	12.81	13.60	13.87	15.81	18.30	13.85	18.29	18.18	4.70E-03	0.78
Quercetin	6.05	5.68	5.26	5.60	5.41	9.16	10.37	9.99	9.31	12.14	3.20E-05	0.55
4-Hydroxybenzoic acid	2.15	2.03	2.05	2.03	1.48	2.99	2.29	2.83	2.44	3.13	4.25E-03	0.71
Ferulic acid	3.32	3.50	3.09	2.83	3.07	2.38	2.18	1.81	2.10	2.55	4.94E-04	1.43
Chlorogenic acid	22.33	2.13	18.10	17.78	9.67	ND	ND	ND	ND	ND		
Neochlorogenic acid	5.47	0.18	3.68	3.06	1.76	ND	ND	ND	ND	ND		
4-Hydroxy-3,5-dimethoxy benzoic acid	0.36	0.57	0.73	1.22	0.64	0.14	0.22	0.63	0.34	1.14	3.90E-01	1.42
Gallic acid	0.37	0.44	0.40	0.40	0.54	0.23	0.34	0.11	0.20	0.26	2.71E-03	1.92
Caffeic acid	0.83	0.92	0.93	0.94	0.71	1.43	1.51	1.41	1.77	1.53	2.86E-05	0.56
trans-Cinnamic acid	1.27	4.13	2.53	1.91	1.28	ND	ND	ND	ND	ND		
3,5-Dimethoxy-4-hydroxycinnamic acid	198.93	208.90	237.40	223.28	236.80	94.98	98.28	103.83	79.45	95.13	4.50E-07	2.34
Apigenin	0.37	0.56	0.50	0.49	0.36	1.14	1.67	1.30	1.26	1.28	1.94E-05	0.34

Protocatechuic acid	1.91	2.04	2.26	2.17	2.05	1.45	1.66	1.23	1.12	1.37	1.89E-04	1.53
Rutin	ND	ND	ND	ND	ND	0.03	0.09	0.11	0.10	0.06		
7-Hydroxycoumarin	0.34	0.21	0.04	0.25	0.13	0.86	0.27	0.26	0.15	0.25	2.70E-01	0.55
Luteolin	5.08	5.38	4.61	4.99	5.33	7.42	8.00	9.31	7.30	8.83	7.35E-05	0.62
Isorhamnetin	5.21	3.16	3.09	4.25	4.33	2.17	2.07	2.06	2.15	2.12	1.47E-03	1.90
6,7-Dihydroxycoumarin	4.89	4.44	4.80	4.74	4.60	3.18	2.99	2.52	2.82	3.01	1.08E-06	1.62
Baicalein	1.06	1.05	1.08	1.05	1.05	1.05	1.06	1.05	1.05	1.08	7.93E-01	1.00
Apigenin 7-glucoside	8.36	8.41	7.90	7.73	7.75	7.79	9.28	8.45	8.79	8.47	1.02E-01	0.94
Isovitexin	57.08	53.15	55.55	51.75	54.18	24.49	25.40	24.21	25.63	25.68	1.63E-09	2.17
Kaempferol-3-O-rutinosid	0.38	0.14	0.17	0.19	0.15	0.76	0.86	1.08	0.97	0.82	1.21E-05	0.23
Astragalin	2.00	1.73	1.63	1.70	1.50	10.67	10.32	8.90	8.80	10.07	3.17E-08	0.18
Syringaldehyde	15.93	15.56	12.99	15.87	17.05	6.12	5.84	5.58	4.29	4.94	8.58E-07	2.89
Homogentisic Acid	4.39	5.52	5.19	5.11	5.31	4.65	4.50	4.67	3.47	4.63	4.36E-02	1.16
Orientin	68.50	77.53	71.23	73.20	79.65	88.73	139.63	120.43	85.68	119.28	8.03E-03	0.67
Glycitein	0.21	0.48	0.45	0.29	0.19	17.73	17.16	14.65	13.75	15.90	3.04E-08	0.02
Genistin	0.08	0.08	0.18	0.07	0.20	0.81	0.80	0.75	0.32	ND	1.48E-03	0.18
Naringin	1.93	2.20	1.96	2.07	1.66	29.28	28.80	26.23	22.59	27.85	3.18E-08	0.07
Eriodictyol	0.11	0.13	0.08	0.11	0.13	0.11	0.10	0.11	0.12	0.13	6.43E-01	0.95
Scopoletin	0.02	0.01	0.01	0.03	0.02	0.05	0.02	0.01	0.04	0.02	3.27E-01	0.70
Chrysoeriol	12.88	11.88	10.42	12.10	11.20	5.01	4.29	5.53	8.89	4.61	1.91E-04	2.07
Luteolin-7-O-β-D-glucopyranoside	12.03	12.46	13.18	13.64	12.88	31.83	35.63	32.00	30.35	34.10	3.28E-08	0.39
Hyperoside	60.18	52.05	58.53	58.13	57.95	82.60	82.13	78.75	75.10	80.83	2.85E-06	0.72
3,4-Dimethoxycinnamic acid	7.85	6.57	6.55	7.04	8.02	7.02	8.55	7.06	5.68	7.17	8.45E-01	1.02

Ellagic acid	8.05	6.63	7.20	7.57	7.26	6.36	6.62	6.39	6.39	6.52	5.71E-03	1.14
Salicylic acid	0.53	0.66	0.41	0.72	0.88	0.26	0.39	0.39	0.39	0.34	8.82E-03	1.82
Isochlorogenic acid A	48.70	14.88	33.83	30.88	27.30	1.33	1.59	1.01	0.90	1.13	5.83E-04	26.14
Isochlorogenic acid B	2.86	18.07	1.94	1.57	29.48	ND	1.98	1.21	0.97	1.48	1.84E-01	7.66
Isochlorogenic acid C	53.88	14.96	37.40	33.80	27.40	ND	ND	ND	ND	ND		
Polydatin	0.19	0.20	0.13	0.18	0.27	0.15	0.21	0.15	0.13	0.17	2.29E-01	1.20
3,4-Dihydroxybenzaldehyde	1.16	1.67	1.12	1.38	1.74	0.59	0.85	0.92	0.71	1.02	4.14E-03	1.72
myricetin	3.26	3.29	3.87	3.64	3.61	3.01	3.07	3.38	2.95	3.41	3.83E-02	1.12

Supplementary Table 5. Absolute/relative quantitative results of 154 polyphenols in *T. chinensis* and *T. ledebouri* extracts.

No	Identification methods	Class	Absolute/relative quantification methods	Code for heatmaps	Metabolite name	Formula	Average contents of *T. chinensis* (μg/g)	Average contents of *T. ledebouri* (μg/g)	Fold (*T. chinensis*/*T. ledebouri*)	p value
**1**	QqQ	Flavonoid aglycones	A	Flavonoid aglycone1	Daidzin	$C_{21}H_{20}O_9$	9.23	2.303	4.01	3.80E-11
**2**	QqQ	Flavonoid aglycones	A	Flavonoid aglycone2	Glycitein	$C_{16}H_{12}O_5$	0.32	15.84	0.02	3.04E-08
**3**	QqQ	Flavonoid aglycones	A	Flavonoid aglycone3	Quercetin	$C_{15}H_{10}O_7$	8.14	16.51	0.49	4.39E-06
**4**	QqQ	Flavonoid aglycones	A	Flavonoid aglycone4	Luteolin	$C_{15}H_{10}O_6$	5.08	8.17	0.62	7.35E-05
**5**	QqQ	Flavonoid aglycones	A	Flavonoid aglycone5	Chrysoeriol	$C_{16}H_{12}O_6$	11.70	5.66	2.07	1.91E-04
**6**	QqQ	Flavonoid aglycones	A	Flavonoid aglycone6	Kaempferol	$C_{15}H_{10}O_6$	1.52	0.98	1.55	3.34E-04
**7**	QqQ	Flavonoid aglycones	A	Flavonoid aglycone7	Myricetin	$C_{15}H_{14}O_9$	3.53	3.16	1.12	3.83E-02
**8**	QqQ	Flavonoid aglycones	A	Flavonoid aglycone8	(-)-Gallocatechin	$C_{15}H_{14}O_7$	1.60	1.79	0.90	4.62E-02
**9**	QqQ	Flavonoid aglycones	A	Flavonoid aglycone9	(-)-Epigallocatechin	$C_{15}H_{14}O_7$	4.21	4.25	0.99	0.43
**10**	QqQ	Flavonoid aglycones	A	Flavonoid aglycone10	Eriodictyol	$C_{15}H_{12}O_6$	0.11	0.12	0.96	0.64

**11**	IDA	Flavonoid aglycones	R	Flavonoid aglycone11	5-Hydroxy-4',7,8-trimethoxyflavone	$C_{18}H_{16}O_6$	24.32	4.13	5.89	1.57E-10
**12**	DIA	Flavonoid aglycones	R	Flavonoid aglycone12	Daidzein	$C_{15}H_{10}O_4$	24.32	4.13	5.89	1.57E-10
**13**	IDA	Flavonoid aglycones	R	Flavonoid aglycone13	4',5-Dihydroxy-3',7-dimethoxyisoflavone	$C_{17}H_{14}O_6$	174.51	51.05	3.42	3.59E-08
**14**	DIA	Flavonoid aglycones	R	Flavonoid aglycone14	Farnisin	$C_{16}H_{12}O_5$	2.13	101.85	0.02	6.89E-08
**15**	IDA	Flavonoid aglycones	R	Flavonoid aglycone15	Diosmetin	$C_{16}H_{12}O_6$	100.58	26.50	3.80	1.95E-06
**16**	IDA	Flavonoid aglycones	R	Flavonoid aglycone16	4',5-Dihydroxy-7,8-dimethoxyflavone	$C_{17}H_{14}O_6$	66.62	20.65	3.23	5.17E-06
17	QqQ	Flavonoid aglycones	A	Flavonoid aglycone17	Apigenin	$C_{15}H_{10}O_5$	0.45	1.33	0.34	1.95E-05
18	QqQ	Flavonoid aglycones	A	Flavonoid aglycone18	Isorhamnetin	$C_{16}H_{12}O_7$	4.01	2.11	1.90	1.48E-03
19	QqQ	Flavonoid aglycones	A	Flavonoid aglycone19	Baicalein	$C_{15}H_{10}O_5$	1.06	1.06	1.00	0.79
20	QqQ	Flavonoid glycosides	A	Flavonoid glycoside1	[a] Astragalin	$C_{21}H_{20}O_{11}$	1.71	9.75	0.176	3.17E-08
21	QqQ	Flavonoid glycosides	A	Flavonoid glycoside2	[a] Naringin	$C_{27}H_{32}O_{14}$	1.97	26.95	0.07	3.18E-08
22	QqQ	Flavonoid glycosides	A	Flavonoid glycoside3	[a] Luteolin-7-O-β-D-glucopyranoside	$C_{21}H_{20}O_{11}$	12.84	32.78	0.39	3.284E-08
**23**	QqQ	Flavonoid glycosides	A	Flavonoid glycoside4	[c] Vitexin	$C_{21}H_{20}O_{10}$	86.12	49.73	1.73	8.18E-07

**24**	QqQ	Flavonoid glycosides	A	Flavonoid glycoside5	[a] Hyperoside	$C_{21}H_{20}O_{12}$	57.37	79.88	0.72	2.85E-06
**25**	QqQ	Flavonoid glycosides	A	Flavonoid glycoside6	[b] Isovitexin	$C_{21}H_{20}O_{10}$	54.34	25.08	2.167	1.63E-09
**26**	QqQ	Flavonoid glycosides	A	Flavonoid glycoside7	[a] Quercitrin	$C_{21}H_{20}O_{11}$	0.49	0.40	1.23	3.76E-05
**27**	QqQ	Flavonoid glycosides	A	Flavonoid glycoside8	[a] Apigenin 7-glucoside	$C_{21}H_{20}O_{10}$	0.53	0.91	0.58	1.11E-04
**28**	QqQ	Flavonoid glycosides	A	Flavonoid glycoside9	[d] Orientin	$C_{21}H_{20}O_{11}$	74.02	110.75	0.67	8.03E-03
29	QqQ	Flavonoid glycosides	A	Flavonoid glycoside10	[a] (-)-Epigallocatechin gallate	$C_{22}H_{18}O_{11}$	3.17	3.22	0.99	0.32
**30**	DIA	Flavonoid glycosides	R	Flavonoid glycoside11	[a] Kaempferol 3-[6''-(3-hydroxy-3-methylglutaryl) hexoside]-7-hexoside	$C_{33}H_{38}O_{20}$	1224.00	1.09	1120.86	8.57E-13
**31**	IDA	Flavonoid glycosides	R	Flavonoid glycoside12	[b] Neocarlinoside	$C_{26}H_{28}O_{15}$	19.43	1.30	14.90	1.16E-12
**32**	DIA	Flavonoid glycosides	R	Flavonoid glycoside13	[c] Kaempferol 3-(6''-ethylglucuronide)	$C_{23}H_{22}O_{12}$	6.58	441.73	0.02	3.37E-12
**33**	DIA	Flavonoid glycosides	R	Flavonoid glycoside14	[a] Unidentified22	$C_{33}H_{38}O_{20}$	41.92	0.02	1925.96	5.03E-12
**34**	IDA	Flavonoid glycosides	R	Flavonoid glycoside15	[b] Unidentified2	$C_{32}H_{30}O_{15}$	2.23	30.02	0.072	2.27E-11
**35**	IDA	Flavonoid glycosides	R	Flavonoid glycoside16	[d] 2''-O-beta-L-galactopyranosylorientin (OGA)	$C_{27}H_{30}O_{16}$	645.34	100.23	6.44	5.77E-11
**36**	DIA	Flavonoid glycosides	R	Flavonoid glycoside17	[c] Kaempferol 7- dihexanoside	$C_{27}H_{30}O_{16}$	0.93	9.67	0.10	8.98E-11

**37**	IDA	Flavonoid glycosides	R	Flavonoid glycoside18	[a] Kaempferol 3-(6''-sinapylglucosyl)-(1->2)- hexoside	$C_{38}H_{40}O_{20}$	84.07	4.64	18.14	1.04E-10
**38**	DIA	Flavonoid glycosides	R	Flavonoid glycoside19	[b] Unidentified19	$C_{23}H_{22}O_{12}$	0.14	12.84	0.01	1.05E-10
**39**	IDA	Flavonoid glycosides	R	Flavonoid glycoside20	[a] 6''-Malonylastragalin	$C_{24}H_{22}O_{14}$	430.68	1016.92	0.42	1.66E-10
**40**	IDA	Flavonoid glycosides	R	Flavonoid glycoside21	[b] Unidentified6	$C_{33}H_{38}O_{19}$	186.59	1.59	117.31	1.91E-10
**41**	DIA	Flavonoid glycosides	R	Flavonoid glycoside22	[a] Quercetin 3-(2'''-feruloyldihexanoside)	$C_{37}H_{38}O_{20}$	0.43	0.05	8.06	3.10E-10
**42**	IDA	Flavonoid glycosides	R	Flavonoid glycoside23	[d] Astragalin 7-deoxyhexoside	$C_{27}H_{30}O_{15}$	0.29	7.88	0.04	3.47E-10
**43**	IDA	Flavonoid glycosides	R	Flavonoid glycoside24	[a] 6''-Malonylcosmosiin	$C_{24}H_{22}O_{13}$	109.80	328.81	0.33	4.72E-10
**44**	IDA	Flavonoid glycosides	R	Flavonoid glycoside25	[d] Kaempferol 3-(6-acetylhexoside)	$C_{23}H_{22}O_{12}$	182.01	467.34	0.39	5.52E-10
**45**	IDA	Flavonoid glycosides	R	Flavonoid glycoside26	[a] Isoorientin 2''-[p-coumaroyl-(->6)-hexoside]	$C_{36}H_{36}O_{18}$	23.34	2.57	9.07	7.04E-10
**46**	DIA	Flavonoid glycosides	R	Flavonoid glycoside27	[a] Delphinidin-3-O-(6-O-feruloyl)-5-dihexanoside	$C_{37}H_{38}O_{20}$	9.10	0.59	15.53	7.94E-10
**47**	IDA	Flavonoid glycosides	R	Flavonoid glycoside28	[a] Betavulgarin hexoside	$C_{23}H_{22}O_{11}$	1.32	40.21	0.03	8.30E-10
**48**	IDA	Flavonoid glycosides	R	Flavonoid glycoside29	[d] Glucosylvitexin	$C_{27}H_{30}O_{15}$	103.79	39.16	2.65	3.67E-09
**49**	IDA	Flavonoid glycosides	R	Flavonoid glycoside30	[a] Quercetin 3-(2'''-caffeyldihexanoside)	$C_{36}H_{36}O_{20}$	1.08	11.24	0.10	4.08E-09

**50**	DIA	Flavonoid glycosides	R	Flavonoid glycoside31	[a] 6'''-O-Sinapoylsaponarin	$C_{38}H_{40}O_{19}$	0.039	1.37	0.03	4.68E-09
**51**	DIA	Flavonoid glycosides	R	Flavonoid glycoside32	[b] Isovitexin 2''-O-(6'''-feruloyl) hexoside	$C_{37}H_{38}O_{18}$	0.07	0.81	0.09	5.88E-09
**52**	IDA	Flavonoid glycosides	R	Flavonoid glycoside33	[a] Quercetin 3-xylosyl-(1->6)- hexoside	$C_{26}H_{28}O_{16}$	39.44	142.69	0.28	6.21E-09
**53**	IDA	Flavonoid glycosides	R	Flavonoid glycoside34	[a] Unidentified7	$C_{56}H_{94}O_{28}$	57.53	105.44	0.55	6.69E-09
**54**	IDA	Flavonoid glycosides	R	Flavonoid glycoside35	[b] Unidentified18	$C_{36}H_{38}O_{19}$	78.58	4.52	17.38	9.89E-09
**55**	DIA	Flavonoid glycosides	R	Flavonoid glycoside36	[d] Kaempferol 3-(6''-acetylhexoside)	$C_{23}H_{22}O_{12}$	1.80	0.50	3.61	1.59E-08
**56**	IDA	Flavonoid glycosides	R	Flavonoid glycoside37	[a] Quercetin 3-(6''-.malonylneohesperidoside)	$C_{30}H_{32}O_{19}$	215.32	72.74	2.96	1.88E-08
**57**	IDA	Flavonoid glycosides	R	Flavonoid glycoside38	[b] 2''-O-D-Arabinopyranosylvitexin 7-methyl ether	$C_{27}H_{30}O_{14}$	2.62	9.69	0.27	2.30E-08
**58**	IDA	Flavonoid glycosides	R	Flavonoid glycoside39	[a] Unidentified5	$C_{35}H_{56}O_{16}$	119.76	44.22	2.71	2.33E-08
**59**	IDA	Flavonoid glycosides	R	Flavonoid glycoside40	[b] Isotectorigenin 4'-hexoside 7-deoxyhexoside	$C_{28}H_{32}O_{15}$	0.09	2.06	0.04	3.06E-08
**60**	DIA	Flavonoid glycosides	R	Flavonoid glycoside41	[b] Isoscoparin 2''-(6-(E)-ferulylhexoside)	$C_{38}H_{41}O_{19}$	6.82	0.28	24.05	3.54E-08
**61**	IDA	Flavonoid glycosides	R	Flavonoid glycoside42	[a] Isorhamnetin 3-(3'''-ferulylrobinobioside)	$C_{38}H_{40}O_{19}$	19.51	7.17	2.72	4.28E-08
**62**	IDA	Flavonoid glycosides	R	Flavonoid glycoside43	[b] Unidentified4	$C_{29}H_{34}O_{16}$	2.35	139.26	0.02	4.99E-08

**63**	DIA	Flavonoid glycosides	R	Flavonoid glycoside44	[c] Margaritene	$C_{28}H_{32}O_{14}$	0.22	8.52	0.03	1.12E-07
**64**	IDA	Flavonoid glycosides	R	Flavonoid glycoside45	[a] Glucosylorientin	$C_{27}H_{30}O_{17}$	21.35	33.465	0.64	1.53E-07
**65**	IDA	Flavonoid glycosides	R	Flavonoid glycoside46	[b] 2''-O-feruloylorientin	$C_{31}H_{28}O_{14}$	5.80	23.47	0.25	1.56E-07
**66**	IDA	Flavonoid glycosides	R	Flavonoid glycoside47	[b] 2''-O-feruloylisoswertiajaponin	$C_{32}H_{30}O_{14}$	7.13	29.20	0.24	1.69E-07
**67**	IDA	Flavonoid glycosides	R	Flavonoid glycoside48	[d] 2''-O-(2''-methylbutyryl)isoswertiajaponin	$C_{27}H_{30}O_{12}$	114.64	254.99	0.45	2.31E-07
**68**	IDA	Flavonoid glycosides	R	Flavonoid glycoside49	[b] 2''-O-feruloylvitexin	$C_{31}H_{28}O_{13}$	11.54	38.88	0.30	2.41E-07
**69**	DIA	Flavonoid glycosides	R	Flavonoid glycoside50	[d] Isoswertisin 2''-O-(2'''-methylbutyrate)	$C_{27}H_{30}O_{11}$	276.39	517.20	0.53	3.66E-07
**70**	DIA	Flavonoid glycosides	R	Flavonoid glycoside51	[a] Quercetin 3-(6''-malonylhexoside)-7-deoxyhexoside	$C_{30}H_{32}O_{19}$	0.11	0.34	0.33	1.15E-06
**71**	DIA	Flavonoid glycosides	R	Flavonoid glycoside52	[a] Luteone 7-hexoside	$C_{26}H_{28}O_{11}$	1.55	2.78	0.56	2.35E-06
**72**	IDA	Flavonoid glycosides	R	Flavonoid glycoside53	[b] 2''-O-(2'''-methylbutyryl)orientin	$C_{26}H_{28}O_{12}$	38.66	85.76	0.45	2.49E-06
**73**	IDA	Flavonoid glycosides	R	Flavonoid glycoside54	[b] Unidentified3	$C_{21}H_{20}O_{11}$	33.95	73.45	0.46	2.81E-06
**74**	IDA	Flavonoid glycosides	R	Flavonoid glycoside55	[d] Isoswertiajaponin	$C_{22}H_{22}O_{11}$	45.09	25.44	1.77	3.52E-06
**75**	DIA	Flavonoid glycosides	R	Flavonoid glycoside56	[b] 4''-Hydroxy-3-'-methoxymaysin	$C_{28}H_{32}O_{14}$	1.71	3.76	0.45	4.34E-06

**76**	DIA	Flavonoid glycosides	R	Flavonoid glycoside57	[b] Unidentified14	$C_{94}H_{110}O_{23}$	1.38	2.98	0.46	6.21E-06
**77**	DIA	Flavonoid glycosides	R	Flavonoid glycoside58	[b] Unidentified21	$C_{26}H_{28}O_{12}$	0.76	1.46	0.53	6.67E-06
**78**	IDA	Flavonoid glycosides	R	Flavonoid glycoside59	[d] Orientin 7-deoxyhexoside	$C_{27}H_{30}O_{15}$	0.14	0.47	0.29	4.95E-06
**79**	IDA	Flavonoid glycosides	R	Flavonoid glycoside60	[b] Isoscoparin 2''-(6-(E)-p-coumaroylhexoside)	$C_{37}H_{38}O_{18}$	8.68	4.44	1.96	2.07E-05
**80**	IDA	Flavonoid glycosides	R	Flavonoid glycoside61	[d] Vitexin 2''-O-(2'''-methylbutyryl)	$C_{26}H_{28}O_{11}$	191.82	290.35	0.66	2.67E-05
**81**	IDA	Flavonoid glycosides	R	Flavonoid glycoside62	[b] 2''-O-vanilloylvitexin	$C_{29}H_{26}O_{13}$	9.96	14.66	0.68	2.16E-04
**82**	DIA	Flavonoid glycosides	R	Flavonoid glycoside63	[b] 2''-O-vanilloylorientin	$C_{29}H_{26}O_{14}$	6.43	9.79	0.67	5.02E-04
**83**	IDA	Flavonoid glycosides	R	Flavonoid glycoside64	[b] 2''-O-(3''',4'''-dimethoxybenzoyl)isoswertiajaponin	$C_{31}H_{30}O_{14}$	23.23	15.48	1.50	1.24E-03
**84**	IDA	Flavonoid glycosides	R	Flavonoid glycoside65	[a] Kaempferol 3-alpha-L-arabinofuranoside	$C_{20}H_{18}O_{10}$	0.36	0.68	0.53	2.43E-03
**85**	DIA	Flavonoid glycosides	R	Flavonoid glycoside66	[a] Quercetin 3-pentoside	$C_{20}H_{18}O_{11}$	0.22	0.35	0.62	8.35E-03
**86**	IDA	Flavonoid glycosides	R	Flavonoid glycoside67	[b] 2''-O-(3''',4'''-dimethoxylbenzoyl) orientin	$C_{30}H_{28}O_{14}$	32.05	41.48	0.77	8.77E-03
**87**	DIA	Flavonoid glycosides	R	Flavonoid glycoside68	[a] Kaempferide 7- hexoside	$C_{22}H_{22}O_{11}$	0.79	0.95	0.83	0.01
**88**	IDA	Flavonoid glycosides	R	Flavonoid glycoside69	[b] Unidentified1	$C_{34}H_{42}O_{18}$	9.65	13.78	0.70	0.02

**89**	DIA	Flavonoid glycosides	R	Flavonoid glycoside70	[a] Unidentified17	$C_{20}H_{18}O_{10}$	0.62	0.80	0.77	0.04
**90**	DIA	Flavonoid glycosides	R	Flavonoid glycoside71	[b] Isoswertisin 3''-O-(2'''-methylbutyrate)	$C_{27}H_{30}O_{11}$	0.19	0.25	0.74	4.98E-02
**91**	DIA	Flavonoid glycosides	R	Flavonoid glycoside72	[a] Unidentified16	$C_{20}H_{18}O_{10}$	1.37	1.62	0.85	0.16
**92**	IDA	Flavonoid glycosides	R	Flavonoid glycoside73	[a] 3',8-Dimethoxyapigenin 7-hexoside	$C_{23}H_{24}O_{12}$	7.97	8.68	0.92	0.20
**93**	IDA	Flavonoid glycosides	R	Flavonoid glycoside74	[b] Unidentified15	$C_{30}H_{28}O_{13}$	52.75	57.79	0.91	0.21
**94**	IDA	Flavonoid glycosides	R	Flavonoid glycoside75	[a] 2''-O-(3''',4'''-dimethoxybenzoyl)isoswertisin	$C_{31}H_{30}O_{13}$	25.60	26.06	0.98	0.78
**95**	QqQ	Phenolic acids	A	Phenolic acid1	Phlorizin	$C_{21}H_{24}O_{10}$	7.36	0.37	19.69	3.52E-10
**96**	QqQ	Phenolic acids	A	Phenolic acid2	3,5-Dimethoxy-4-hydroxycinnamic acid	$C_{11}H_{12}O_5$	221.06	94.33	2.34	4.50E-07
**97**	QqQ	Phenolic acids	A	Phenolic acid3	Syringaldehyde	$C_9H_{10}O_4$	15.48	5.35	2.89	8.58E-07
**98**	QqQ	Phenolic acids	A	Phenolic acid4	6,7-Dihydroxycoumarin	$C_9H_6O_4$	4.69	2.90	1.62	1.09E-06
**99**	QqQ	Phenolic acids	A	Phenolic acid5	Caffeic acid	$C_9H_8O_4$	0.86	1.53	0.57	2.86E-05
**100**	QqQ	Phenolic acids	A	Phenolic acid6	Protocatechuic acid	$C_7H_6O_4$	2.09	1.37	1.53	1.89E-04
**101**	QqQ	Phenolic acids	A	Phenolic acid7	Ferulic acid	$C_{10}H_{10}O_4$	3.16	2.20	1.43	4.94E-04

**102**	QqQ	Phenolic acids	A	Phenolic acid8	Isochlorogenic acid A	$C_{25}H_{24}O_{12}$	31.12	1.19	26.14	1.79E-06
**103**	QqQ	Phenolic acids	A	Phenolic acid9	3,4-Dihydroxybenzaldehyde	$C_7H_6O_3$	1.41	0.82	1.72	4.14E-03
**104**	QqQ	Phenolic acids	A	Phenolic acid10	4-Hydroxybenzoic acid	$C_7H_6O_3$	1.95	2.74	0.71	4.25E-03
**105**	QqQ	Phenolic acids	A	Phenolic acid11	Chlorogenic acid	$C_{16}H_{18}O_9$	0.73	0.02	29.87	4.44E-03
**106**	QqQ	Phenolic acids	A	Phenolic acid12	Vanillic acid	$C_8H_8O_4$	13.10	16.89	0.78	4.70E-03
**107**	QqQ	Phenolic acids	A	Phenolic acid13	Ellagic acid	$C_{14}H_6O_8$	7.34	6.46	1.14	5.71E-03
**108**	QqQ	Phenolic acids	A	Phenolic acid14	Salicylic acid	$C_7H_6O_3$	0.64	0.35	1.82	8.82E-03
**109**	QqQ	Phenolic acids	A	Phenolic acid15	trans-Cinnamic acid	$C_9H_8O_2$	2.22	0.47	4.77	0.02
**110**	QqQ	Phenolic acids	A	Phenolic acid16	trans-4-Hydroxycinnamic acid	$C_9H_8O_3$	2.97	4.14	0.72	0.03
**111**	QqQ	Phenolic acids	A	Phenolic acid17	4-O-Caffeoylquinic acid	$C_{16}H_{18}O_9$	0.89	0		0.03
**112**	QqQ	Phenolic acids	A	Phenolic acid18	Homogentisic acid	$C_8H_8O_4$	5.10	4.38	1.16	0.04
**113**	QqQ	Phenolic acids	A	Phenolic acid19	Isochlorogenic acid B	$C_{25}H_{24}O_{12}$	10.78	1.41	7.66	0.13
**114**	QqQ	Phenolic acids	A	Phenolic acid20	Polydatin	$C_{20}H_{22}O_8$	0.20	0.16	1.20	0.23

**115**	QqQ	Phenolic acids	A	Phenolic acid21	7-Hydroxycoumarin	$C_9H_6O_3$	0.20	0.36	0.55	0.27
**116**	QqQ	Phenolic acids	A	Phenolic acid22	Scopoletin	$C_{10}H_8O_4$	0.02	0.03	0.70	0.33
**117**	QqQ	Phenolic acids	A	Phenolic acid23	4-Hydroxy-3,5-dimethoxy benzoic acid	$C_9H_{10}O_5$	0.71	0.50	1.42	0.39
**118**	QqQ	Phenolic acids	A	Phenolic acid24	3,4-Dimethoxycinnamic acid	$C_{11}H_{12}O_4$	7.21	7.09	1.02	0.85
**119**	QqQ	Phenolic acids	A	Phenolic acid25	Butein	$C_{15}H_{12}O_5$	0.04	0.04	0.97	0.92
**120**	QqQ	Phenolic acids	A	Phenolic acid26	Isochlorogenic acid C	$C_{25}H_{24}O_{12}$	35.49	0.15	240.63	8.19E-11
**121**	IDA	Phenolic acids	R	Phenolic acid27	Trollioside	$C_{19}H_{26}O_9$	264.36	10.02	26.39	3.26E-11
**122**	IDA	Phenolic acids	R	Phenolic acid28	Vanillylmandelic acid	$C_9H_{10}O_5$	5.08	27.95	0.18	5.81E-11
**123**	IDA	Phenolic acids	R	Phenolic acid29	Unidentified13	$C_{19}H_{26}O_{10}$	58.65	3.40	17.27	7.15E-11
**124**	DIA	Phenolic acids	R	Phenolic acid30	cis-Sinapic acid	$C_{11}H_{12}O_5$	7.77	1.20	3.89	1.61E-10
**125**	IDA	Phenolic acids	R	Phenolic acid31	Salviaflaside	$C_{24}H_{26}O_{13}$	17.01	3.350	5.08	2.64E-10
**126**	IDA	Phenolic acids	R	Phenolic acid32	Sinaptic acid	$C_{11}H_{12}O_5$	78.95	16.04	4.9209	2.17E-09
**127**	IDA	Phenolic acids	R	Phenolic acid33	Veratric acid	$C_9H_{10}O_4$	19.67	5.05	3.90	6.00E-09

**128**	IDA	Phenolic acids	R	Phenolic acid34	Unidentified11	$C_{36}H_{38}O_{18}$	35.87	2.11	16.98	1.01E-08
**129**	IDA	Phenolic acids	R	Phenolic acid35	Hydroxytyrosol 1-O- hexoside	$C_{14}H_{20}O_{8}$	149.34	231.19	0.65	3.00E-08
**130**	IDA	Phenolic acids	R	Phenolic acid36	Vanillic acid 4-O-glucuronide	$C_{14}H_{16}O_{10}$	1.24	3.23	0.38	1.37E-07
**131**	DIA	Phenolic acids	R	Phenolic acid37	4-Hydroxy-3,5-dimethoxybenzaldehyde	$C_{9}H_{10}O_{4}$	0.75	0.10	7.47	1.46E-07
**132**	IDA	Phenolic acids	R	Phenolic acid38	Vanillylamine	$C_{8}H_{11}NO_{2}$	110.06	58.77	1.87	1.51E-07
**133**	IDA	Phenolic acids	R	Phenolic acid39	3-(6-hydroxy-7-methoxy-2H-1,3-benzodioxol-5-yl)propanoic acid	$C_{11}H_{12}O_{6}$	232.04	120.46	1.93	6.02E-07
**134**	IDA	Phenolic acids	R	Phenolic acid40	4-Hydroxycinnamic acid	$C_{9}H_{8}O_{3}$	0.32	0.13	2.47	1.16E-06
**135**	DIA	Phenolic acids	R	Phenolic acid41	(2R,3S)-Piscidic acid	$C_{11}H_{12}O_{7}$	8.70	21.79	0.40	3.68E-06
**136**	IDA	Phenolic acids	R	Phenolic acid42	Fukiic acid	$C_{11}H_{12}O_{8}$	14.99	38.51	0.39	4.93E-06
**137**	IDA	Phenolic acids	R	Phenolic acid43	Hydroxytyrosol 3'-glucuronide	$C_{14}H_{18}O_{9}$	28.97	57.96	0.50	9.02E-06
**138**	IDA	Phenolic acids	R	Phenolic acid44	Unidentified20	$C_{19}H_{26}O_{10}$	1.54	0.26	5.84	9.13E-06
**139**	IDA	Phenolic acids	R	Phenolic acid45	Monotropein	$C_{16}H_{22}O_{11}$	90.61	158.44	0.57	6.13E-05
**140**	IDA	Phenolic acids	R	Phenolic acid46	Glucoobtusifolin	$C_{22}H_{22}O_{10}$	10.85	6.90	1.57	9.66E-05

**141**	IDA	Phenolic acids	R	Phenolic acid47	Unidentified23	$C_{11}H_{12}O_7$	328.84	230.78	1.43	1.24E-04
**142**	DIA	Phenolic acids	R	Phenolic acid48	4,5-Di-O-caffeoylquinic acid	$C_{25}H_{24}O_{12}$	0.07	0.02	4.32	2.40E-03
**143**	IDA	Phenolic acids	R	Phenolic acid49	Unidentified12	$C_{22}H_{38}O_{12}$	18.19	23.37	0.78	2.47E-03
**144**	QqQ	Phenolic acids	A	Phenolic acid50	Gallic acid	$C_7H_6O_5$	0.43	0.22	1.92	2.71E-03
**145**	IDA	Phenolic acids	R	Phenolic acid51	3,5-Di-O-caffeoylquinic acid	$C_{25}H_{24}O_{12}$	1.43	0.21	6.97	3.55E-03
**146**	DIA	Phenolic acids	R	Phenolic acid52	Vanillin	$C_8H_8O_3$	0.43	0.32	1.36	7.21E-03
**147**	DIA	Phenolic acids	R	Phenolic acid53	Unidentified10	$C_{27}H_{24}O_{18}$	1.01	0.79	1.29	1.71E-02
**148**	IDA	Phenolic acids	R	Phenolic acid54	2-Pyrocatechuic acid	$C_7H_6O_4$	0.46	0.59	0.79	2.46E-02
**149**	IDA	Phenolic acids	R	Phenolic acid55	Ascorbic acid	$C_6H_8O_6$	17.87	20.76	0.86	0.10
**150**	IDA	Phenolic acids	R	Phenolic acid56	Quercetin 3-lathyroside	$C_{26}H_{28}O_{16}$	0.85	0.94	0.904	0.17
**151**	DIA	Phenolic acids	R	Phenolic acid57	Shikimic acid	$C_7H_{10}O_5$	5.14	4.06	1.27	0.43
**152**	IDA	Phenolic acids	R	Phenolic acid58	Unidentified9	$C_{58}H_{94}O_{29}$	25.09	25.80	0.97	0.59

**153**	IDA	Phenolic acids	R	Phenolic acid59	(±)-threo-1-(p-Hydroxyphenyl)propylene glycol 4'-hexoside	$C_{15}H_{22}O_8$	118.85	32.96	3.61	1.74E-10
**154**	IDA	Phenolic acids	R	Phenolic acid60	Unidentified8	$C_{16}H_{24}O_{10}$	196.01	56.64	3.46	2.82E-10

Note: A, absolute quantification; R, relative quantification

Compared with reference 31, newly detected in this work, [a] flavone O-glycosides, [b] flavone C-glycosides; detected in both work for [c] flavone O-glycosides and [d] flavone C-glycosides
